# Psychosocial and demographic predictors of adherence and non-adherence to health advice accompanying air quality warning systems: a systematic review

**DOI:** 10.1186/s12940-017-0307-4

**Published:** 2017-09-22

**Authors:** Donatella D’Antoni, Louise Smith, Vivian Auyeung, John Weinman

**Affiliations:** 1 King’s College London, Institute of Pharmaceutical Sciences, 150 Stamford Street, London, SE1 9NH UK; 2 King’s College London, Department of Psychological Medicine, De Crespigny Park, London, UK

**Keywords:** Systematic review, Adherence, Behaviour change, Psychosocial factors, Air quality alerts

## Abstract

**Background:**

Although evidence shows that poor air quality can harm human health, we have a limited understanding about the behavioural impact of air quality forecasts. Our aim was to understand to what extent air quality warning systems influence protective behaviours in the general public, and to identify the demographic and psychosocial factors associated with adherence and non-adherence to the health advice accompanying these warnings.

**Method:**

In August 2016 literature was systematically reviewed to find studies assessing intended or actual adherence to health advice accompanying air quality warning systems, and encouraging people to reduce exposure to air pollution. Predictors of adherence to the health advice and/or self-reported reasons for adherence or non-adherence were also systematically reviewed. Studies were included only if they involved participants who were using or were aware of these warning systems. Studies investigating only protective behaviours due to subjective perception of bad air quality alone were excluded. The results were narratively synthesised and discussed within the COM-B theoretical framework.

**Results:**

Twenty-one studies were included in the review: seventeen investigated actual adherence; three investigated intended adherence; one assessed both. Actual adherence to the advice to reduce or reschedule outdoor activities during poor air quality episodes ranged from 9.7% to 57% (*Median* = 31%), whereas adherence to a wider range of protective behaviours (e.g. avoiding busy roads, taking preventative medication) ranged from 17.7% to 98.1% (*Median* = 46%). Demographic factors did not consistently predict adherence. However, several psychosocial facilitators of adherence were identified. These include knowledge on where to check air quality indices, beliefs that one’s symptoms were due to air pollution, perceived severity of air pollution, and receiving advice from health care professionals. Barriers to adherence included: lack of understanding of the indices, being exposed to health messages that reduced both concern about air pollution and perceived susceptibility, as well as perceived lack of self-efficacy/locus of control, reliance on sensory cues and lack of time.

**Conclusion:**

We found frequent suboptimal adherence rates to health advice accompanying air quality alerts. Several psychosocial facilitators and barriers of adherence were identified. To maximise their health effects, health advice needs to target these specific psychosocial factors.

**Electronic supplementary material:**

The online version of this article (10.1186/s12940-017-0307-4) contains supplementary material, which is available to authorized users.

## Background

In 2014 a World Health Organisation (WHO) report revealed that around 3.7 million people had died prematurely in the world in 2012 as a result of exposure to ambient air pollution. These deaths were attributed to specific diseases such as heart disease, stroke, chronic obstructive pulmonary disease (COPD), lung cancer and acute respiratory infections in children [1]. Findings from epidemiological and toxicological studies have highlighted negative short- and long-term effects of air pollution on both premature mortality and morbidity from respiratory and cardiovascular disease, following both short-term and chronic exposure. Recent studies are also investigating the potential for particulate air pollution to negatively impact birth outcomes, cognitive function and diabetes (for an overview, see [2]). To complicate matters further, there is little evidence of what constitutes a safe level of exposure or what is the exact threshold below which no adverse health effects occur [2]. In this context, it has been recommended to raise awareness amongst the general population, and in particular amongst individuals who are more susceptible to experience symptoms (e.g. due to lung or heart problems), about the health impact of air pollution, and to provide advice on how to reduce exposure [3]. However, research indicates a lack of awareness among the public about the links between air pollution and illness, as well as a lack of understanding of air quality information (e.g. [4–6]). Moreover, it is now clear that the traditional strategy of simply informing people about high pollution episodes is not effective (e.g. [7]). In the current paper we are presenting the results of the systematic review we carried out to understand the extent to which air quality warning systems influence protective behaviours in the general public, and to identify the factors associated with adherence and non-adherence to health advice received through these systems.

Environmental behaviour is complex and is better understood if we consider a combination of its multiple determinants, including attitudes and perceptions, as well as personal capabilities, context and habits [8]. In line with this, the COM-B framework, developed from existing theories of behaviour change [9], proposes that to better understand the determinants of health behaviour we should consider the interactions existing between capability (C), opportunity (O) and motivation (M), where individual, group and environmental determinants are equally considered in controlling behaviours (B). The COM-B seems to offer a comprehensive framework, where ‘capability’ is defined as the individual’s psychological (e.g. knowledge, understanding) and physical capacity to engage in the targeted activity, ‘opportunity’ refers to all the external factors that make the behaviour possible or prompt it, and ‘motivation’ to the mental processes that energise and direct behaviour, including beliefs, attitudes and habitual processes and emotional responses. In the present review, we are going to use this framework to discuss the factors identified as facilitators or barriers of adherence to air quality warning systems. The rationale is based on the ability of the COM-B to consider a wide range of predictors of adherence, which can also guide in the identification of behaviour change interventions [10, 11] aiming at improving the effectiveness of risk communications. As others have stressed [3], we have a very limited knowledge about whether the existing air quality alerts are effective in encouraging people to take protective behaviour to reduce exposure to air pollution. Our results have the potential to inform local authorities and environmental agencies regarding how to communicate to the general public in order to encourage them to take protective actions during days of poor air quality. Information about air quality can be reported via daily reports on news media, environmental protection agencies webpages, air quality forecasts and quasi-real time alerts sent when air quality reaches specific levels, for instance via social media, smartphone applications, email alerts, and text messages. To make it easier to understand the levels of air quality, information services adopt different colour banding and index values. In the UK, information about the air quality levels is provided by the Department for Environment Food and Rural Affairs (DEFRA) in the form of a national air quality index (AQI), together with health advice about how to reduce exposure. There is variability amongst countries in the number of bandings and index values used [12]. For instance, the US [13] adopts an AQI that uses a scale from 0 to 500, where the higher the air quality value, the greater the level of air pollution. This scale presents 6 bandings of health concern (Good, Moderate, Unhealthy for Sensitive Groups, Unhealthy, Very Unhealthy and Hazardous) and considers pollutants such as carbon monoxide (CO), nitrogen dioxide (NO_2_), ozone (O_3_), fine particulate matters PM_2.5_ and PM_10_, and sulphur dioxide (SO_2_). On the other hand, the UK [14] uses a 10-point scale with 10 bandings (although only 4 named bandings, see Table 1) and considers pollutants such as NO_2_, O_3_, PM_2.5_, PM_10_, SO_2_. The type of health advice can also differ depending on the specific country. For instance, in the UK the AQI provides, for each air pollution banding, separate advice for the general population and for individuals who are at greater risk of experiencing symptoms, such as people with pre-existing heart or lung conditions (see Table 1), whilst this is not the case in the US.

**Table 1:** Health advice accompanying the UK AQI

Air pollution Banding	Value	Accompanying health messages for at-risk groups and the general population	
		At-risk individuals^a^	General population
Low	1–3	*Enjoy* your usual outdoor activities.	*Enjoy* your usual outdoor activities.
Moderate	4–6	Adults and children with lung problems, and adults with heart problems, *who experience symptoms*, should *consider reducing* strenuous physical activity, particularly outdoors.	*Enjoy* your usual outdoor activities.
High	7–9	Adults and children with lung problems, and adults with heart problems, should *reduce* strenuous physical exertion, particularly outdoors, and particularly if they experience symptoms. People with asthma may find they need to use their reliever inhaler more often. Older people should also *reduce* physical exertion	Anyone experiencing discomfort such as sore eyes, cough or sore throat should *consider reducing* activity, particularly outdoors.
Very High	10	Adults and children with lung problems, adults with heart problems, and older people, should *avoid* strenuous physical activity. People with asthma may find they need to use their reliever inhaler more often.	*Reduce* physical exertion, particularly outdoors, especially if you experience symptoms such as cough or sore throat.

^a^Adults and children with heart or lung problems are at greater risk of symptoms

Italicised word in original [6]

As previously described by Skov and colleagues [15], there are mainly two types of relevant health behaviours in relation to air quality alerts: actions aimed at reducing air pollution and actions aimed at self-protection from air pollution. The present systematic review focused exclusively on the latter. In this context, researchers have considered different measures of adherence such as self-reports of protective behaviours, direct observation of avoidant behaviours during air pollution episodes (e.g. reduction in park attendance, changes in minutes spent outdoors, percentages of facemasks sold), and indirect indicators of avoidant behaviours such as reduction in emergency service admissions due to respiratory symptoms triggered by exposure to air pollution. The aim of this systematic review was to understand the extent to which air quality warning systems influence protective behaviours in the general public, and to identify the demographic and psychosocial factors associated with adherence and non-adherence to health advice received through these systems. To improve the validity of our results, we included only studies where participants were either users of some sort of air quality warning system or were explicitly asked to report their behaviours in relation to hearing or reading official air quality information.

## Methodology

The present review is reported in accordance with the Preferred Reporting Items for Systematic reviews and Meta-Analysis (PRISMA) guidelines [16], using systematic methods to identify and select studies, and assess their risk of bias.

### Search strategy

We searched electronic databases such as Web of Science Core Collection, OVID (Global Health 1973 to 2016 week 18), PsycINFO (1806 to May 2016), Social Policy and Practice (2016), Embase (1947 to May 2016), Ovid MEDLINE(R) (1946 to present), Science Direct, Scopus, Pubmed, CINAHL in August 2016. In addition, OpenGrey.eu, EThos, and Google were used to identify relevant unpublished studies and reports (e.g. governmental reports). No date limit or study type limit were applied, however papers published only as abstracts were excluded. We only searched for literature written in English. The search strategy combined terms related to air quality alerts (i.e. ‘air quality alert’, ‘air quality index’, ‘air quality advisories’, ‘smog alert’), and adherent and protective behaviours (i.e. ‘adherence’, ‘compliance’, ‘health behaviour’, ‘risk reduction’, ‘public response’). The search was conducted to make sure that both the general public and vulnerable population (e.g. asthmatics) were included. The Additional file 1 shows the full search strategy for the majority of the databases. Further articles were included through manually screening reference lists of relevant articles and reports.

### Inclusion criteria

The inclusion criteria were based on the Participants, Interventions, Outcomes, and Setting -PI(C)OS - approach in the PRISMA guidelines. Studies were included if they met the following criteria:

i) Participants: people who read or heard of air quality reports, alerts, indices or other sources of health information related to air quality (e.g. users of air quality warning systems, people familiar with air quality forecasts). Participants could be drawn from the general public, patient groups or specific occupational groups.
ii) Interventions: exposure to information about air quality and/or health advice associated with air quality levels, including information related to actual and/or hypothetical levels of air pollution.
iii) Outcomes/Predictors: Actual and/or intended adherence/behaviour change in response to health advice accompanying air quality warning systems and encouraging protective behaviour against air pollution,
  AND/OR.
  Predictors of, and/or self-reported reasons for adherence or non-adherence to health advice associated with air quality information.
iv) Study reporting: All study designs, aside from those published only as editorials or abstracts, were included.

Studies were excluded if they met the following criteria:

i) Were based on the assumption that the respondents were aware of air quality alerts during alert days (i.e. every time an alert was issued in a specific area), but did not collect evidence of this;
ii) Analysed behaviour change in response to air quality as driven by people’s own perception of air quality, without the involvement of any official information;
iii) Analysed only information-seeking behaviour and/or frequency of access of air quality information services, without investigating behavioural changes in response to this activity
iv) Investigated pro-environmental behaviours only (e.g., reducing energy consumption, or avoiding driving the car during pollution episodes to reduce contribution to air pollution). However, when a measured behaviour change (e.g., changing means of transport) was classified as potentially both protective behaviour and pro-environmental behaviour, these measures were included in the review.

### Data extraction and procedure

A standardised form was used to extract data from each study, including details relating to the author, date of publication, country, type of publication, study design, sample size, cohort, type of measure of adherence, adherence rates, together with self-reported reasons for, and predictors of adherence and/or non-adherence. In addition, as recommended by Dombrowski et al. [17] when testing behaviour change interventions, we recorded details on type of air quality information service, type of health recommendation, and delivery format (including details on message provider, target population, channel used, and whether the message was individually tailored or not). Authors were contacted when additional information was needed in order to decide on their inclusion. Since one author could not be contacted, we excluded their study [18] as we did not have enough data to decide whether or not they fully met our inclusion/exclusion criteria.

### Risk of bias

Similarly to previous systematic reviews [19, 20], risk of bias was determined according to an adaptation of the Scottish Intercollegiate Guidelines Network (SIGN) critical appraisal methodology checklist for cohort studies [21], and supplemented by relevant items from the Cochrane Collaboration’s Risk of Bias tool [22]. Each article was assessed for presence of risk of bias by two independent reviewers. Any discrepancies were resolved through discussion. The tool used in the assessment included four criteria (selection bias, detection bias, reporting bias and other bias) (Additional file 2: Table S1). Each of the 21 articles was rated on the four criteria as ‘high’, ‘moderate’ or ‘low’ risk, depending on characteristics reported in the study. For a criterion to be rated as having a ‘low risk of bias’, all of its components had to be rated as low risk. A criterion was rated as having a ‘moderate risk’ when the presence of a possible bias was identified only for a minority of its components. In all other cases, ‘high risk’ ratings were used. Moreover, as the vast majority of the included studies used non-validated adherence measures, we decided to assess also their ‘face-validity’. When a study used an outcome measure deemed to have at least good face-validity, and had no other detection biases, the risk of detection bias for that study was rated as ‘moderate’.

### Data synthesis

Where possible, we grouped study results together depending on whether they related to actual or intended adherence, and whether they assessed only reduction or postponement of outdoor activities as outcome measures or other protective behaviours as well. Results are also presented separately for studies focusing on actual adherence to health warnings sent via personal or non-personal delivery channels, and for qualitative data. A meta-analysis of the data was not planned, based on our anticipation of a very heterogeneous literature. We decided instead to carry out a narrative synthesis of the data [23], with the intention of providing a comprehensive list of predictors of, and self-reported reasons for adherence and non-adherence to air quality information services, using the COM-B framework. For each study, we reported the overall adherence rates and also, where reported by individual studies, we compared adherence rates in subgroups of healthy and vulnerable respondents. Where studies reported only rates for these subgroups, for consistency, we calculated the overall adherence rates based on the overall sample used for the relative analyses. Where reported by individual studies, numerical data for significant predictors (i.e. associated with a *p*-value <0.05) are provided in Additional file 3: Table S2.

## Results

### Studies characteristics

**Fig. 1:**
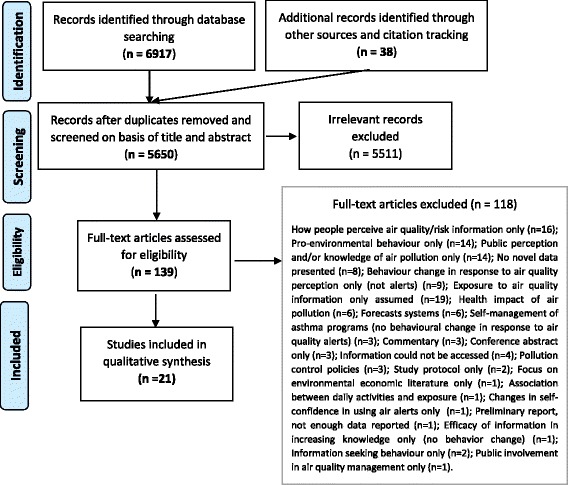
PRISMA flow diagram with literature search. The last search was run on 9 August 2016

Figure 1 illustrates the results of our literature search. We identified 6917 citations through our online database search. A further 38 records were independently identified through other sources and using reference lists of relevant papers and reports. For two of the included studies, additional information was obtained from the author [24, 25]. After removing duplicate records, a total of 5650 records were screened based on their title and abstract, and 5511 irrelevant records were excluded, leaving 139 articles for full-text screening. Twenty-one studies fully met our inclusion criteria and were included in this review. Reasons for exclusion are reported in Fig. 1.

Table 2 provides a summary of the studies included in this review. Ten studies were conducted in the US [25–34], whereas five were conducted in the UK [24, 35–38], four in Canada [39–42], one in Hong Kong [42], and one in Denmark [15]. Data collection covers the period from 1982 to 2016. The vast majority of the included studies were cross-sectional surveys [15, 24–26, 29–35, 37, 38, 40, 42, 43], whereas the remaining studies adopted a cross-sectional quasi-experimental design with quasi-randomisation and a control group [27, 28], a quasi-experimental design using linked data [36], and a one-group pre- post-test design [39]. Although there was only one qualitative study [41], some studies also collected qualitative data in the form of self-reported reasons for adherence and/or non-adherence [24, 30, 40, 42]. The studies included samples of general public, service users of air alert systems, asthmatic patients and people with other respiratory and/or heart conditions, elderly, people who spend most of their time working in busy streets, communities involved in wildfire events, parents of healthy and parents of asthmatic children, and health care professionals. Three studies showed hypothetical air pollution scenarios to their participants [27, 28, 37], one used both hypothetical and real scenarios [15].

**Table 2:** Data extraction showing methods used in the included studies

Author	Publication	Study design	Setting	Sample size	Sample	Sample demographics
Evans et al. [26]	JA	CSS	Los Angeles Twice: Spring-Autumn 1982	*N* = 1002 (~*n* = 500 participants in each period)	Residents of Los Angeles	Not reported. The sample compared favourably to the 1980 U.S. census, with <5% error in gender and ethnic distribution (with more females and Caucasians) and <1% error in age)
Hartill [24]	R	CSS	Southampton, UK 2011, 2014	In 2011, *N* = 74; in 2014, *n* = 52	Air Alert system users	Not reported
Johnson [27]	JA	QES	Philadelphia Jul 1998- Feb 1999	N ~ 1000	People awaiting for jury duty in city courts	Age = 20–81 range; Gender: female = 60% (*N* = 606); Ethnicity: White female = (*n* = 286), non-white females (*n* = 320)
Johnson [28]	JA	QES	Philadelphia Jul 1998- Feb 1999	N ~ 1000	People awaiting for jury duty in city courts	Age (M) = 42.8 years (SD = 12.2, range 18–81); Gender: female = 60%; Ethnicity (White = 49%, African America *n* = 44%, Asia*n* = 3%); Health: self-reported respiratory problem = 29%; had children at home with respiratory disease = 20%.
Kentucky Health Issues Poll [34]	R	CSS	Kentucky Sep -Oct 2012	1680	Kentucky residents	Age (groups) =18–29 years = 22% (*n* = 366); 30–45 = 32% (*n* = 531); 46–64 = 29% (*n* = 481); ≥ 65 = 17% (*n* = 272); Gender: female 52% (*n* = 875); Ethnicity: African American =7% (*n* = 115); White = 88% (*n* = 1486); Health: Self-reported Chronic Disease: (*n* = 902)
Kilbane-Dawe et al. [35]	WP	CSS	Croydon, UK Jun 2006 & Sep 2006	*N* = 168 users completed 382 questionnaires	Service users of the alert system	Not reported
Licskai et al. [39]	JA	Pre-Post T	Windsor, Ontario Jul & Sep 2010	*N* = 22	Convenience sample of adult users of the Primary Care Asthma Program	Age (M) = 47 years (SD = 12); Gender: female = 82% (*n* = 18); Health: All asthmatics; Currently smoking: 18% (n = 4). Taking controller medication: (82%) n = 18; ICS alone (14%) n = 3; Combined therapy (ICS + LABA) = (68%) *n* = 15; Taking rescue medication (past 2 weeks) (68%) n = 15; Rescue medication dose/day, mean (±SD) = 0.92 (±1.53).
Lyons et al. [36]	JA	QES, R & P	South Wales May 2012- Aug 2014	*N* = 179 intervention group (IG) and *N* = 1214 for control group (CG)	Asthmatics, COPD or coronary heart disease patients from local general practices	Age (Modal age group) = 65–74 years in the IG = 33.5% (*n* = 60) and CG = 18.1% (*n* = 220), χ2 = 46.3, *p* < 0.001; Gender: female = IG = 51.4% (*n* = 92), CG = 48% (*n* = 583); Health: all with respiratory condition
Mak, et al. [43]	CP	CSS	Hong Kong 2009	Total *N* = 846. *n* = 216 (from general public) & *n* = 640 (from people working on busy roads)	General public	Not reported
Mansfield et al. [33]	R	CSS	United States Aug 3, 2000 to Sep 13, 2000	*N* = 6106 (used for analyses)	General public	Age (M) = 45 (*n* = 53 missing); Gender: male = 49% (*n* = 38 missing); Ethnicity: White = 81%; Black/ African American =11%, American Indian =2%, Asian =3%, Other =4%; (*n* = 338 missing); Health: Self-reported poor health =3%; (n = 22 missing).
Laube [38]	T	CSS	London, UK Jun-Jul 2012	*N* = 70	LondonAir mobile phone app users	Age (M) = 41.11 years (range 18–67 years); Gender: female = 32.9% (*n* = 23); Health: self-reported respiratory/coronary problems = 44.3%
McDermott et al. [25]	JA	CSS	Salt Lake, US Jan & Sep 2003	*N* = 208 used for analyses. *n* = 110 (parents from asthma cohort), *n* = 98 (parents from non-asthma cohort)	Parents taking their child to a paediatric asthma specialty clinic or attending a general paediatrics clinic	Child Age (M) = 10 years (asthmatics) 9 years (non-asthmatics), *p* = 0.21; Health: in the asthma cohort, 88% (96/110) had a child using a controller inhaler, and 64% (70) reported that their child had a prior A&E visit or hospitalization for asthma; 25% were classified as having moderate or severe asthma (based on NIH Guidelines).
Radisic, et al. [41]	JA	QS	Hamilton, Ontario, Oct 2012 - Apr 2015	*N* = 50 (n = 6 interview participants (Int.); n = 44 focus group participant (FG))	Overall = 6 health care providers, 16 parents, 13 elderly, 15 with respiratory conditions.	Age (groups) (FG): 18–34 = 29% (*n* = 13), 35–54 = 11% (n = 5), ≥55 = 60% (*n* = 26); Gender: female (Int.) = 83% (n = 5), in the FG = 77% (*n* = 34); Health (Int.): with respiratorycondition = 33.3% (n = 2); FG: with respiratory condition: 34% (n = 15).
Radisic et al. [40]	JA	CSS	Hamilton, Ontario, Jun - Oct 2012	*N* = 707	General public	Age (groups) 18–34 = 25% (*n* = 174); 35–54 = 45% (*n* = 318); ≥ 55 = 30% (*n* = 215); Gender: female *n* = 479 (68%); missing = 3% (*n* = 24); Health: self-reported respiratory condition: 25% (*n* = 179); Missing: 1% (n = 3); cardiovascular condition: 9% (*n* = 63); Missing: 1% (n = 8).
Reams et al. [29]	JA	CSS	Upper Industrial CorridorLouisiana 2011	*N* = 64	Attendees of 3 East Baton Rouge Metropolitan Council meetings	Age: ≥50 age group = 80%; Gender: female = 54%.
Semenza et al. [30]	JA	CSS	Portland, OR and Houston, TX, Summers 2005–2006	*N* = 1962 (*n* = 1254 were residents of Portland, and *n* = 708 residents of Houston)	Residents of two US cities	(Data reported for Portland and Houston respectively) Age (M) = 52 years (SD =16.08) & 48.4 (SD = 16.28), *p* = .001; Gender: female = 63.9% & 63.6%, *p* = 0.659; Ethnicity: White 90.7% & 60.5%; Black or African American 1.6% & 17.2%; Asian 2.4% & 5.4%; Hispanic/Latino 2.6% & 13.9%; Other 2.7% & 3% (*p* < 0.0010); Health: 16.6% & 20.7% (*p* = .021) had someone in the household with a medical condition.
Skov et al. [15]	JA	CSS	Copenhagen area, Apr 1989	*N* = 877 (considered for analysis)	Adult residents in Copenhagen (also people from a league for lung-diseased patients)	Age (groups) = above 55 years (in the patient league sample) = 75%; above 55 years (in the population sample) = 27%; Health: people with lung disease: *n* = 192.
Smallbone [37]	R	CSS	London and other locations in the UK 2010	*N* = 411 online surveys. Response rates ranged from 259 to 411	General public	Age (*n* = 396) = age group ≤24 = 8% (n = 31); ≥65 age = 7% (n = 27). Gender: female = 62% (*n* = 244) (*t* = 24.9, *p* < 0.01). Ethnicity: white/white British = 92% (*n* = 349/381); Asian, black/black British: 1% each, Other: 6%. Health (*n* = 131 provided 150 responses): 60% (n = 90) reported asthma, 29% other respiratory illness, 11% heart condition.
Stieb et al. [42]	JA	CSS	Four areas in Canada 1994	*N* = 1474 (New Brunswick *n* = 405; Toronto *n* = 413; Norfolk *n* = 256; Vancouver *n* = 400)	Residents of four areas of Canada, following a smog forecast	Age (M) = New Brunswick =45 years (SD = 17); Toronto =42 (17); Norfolk =46 (16); Vancouver =41 (15); Gender: female: New Brunswick: 61%; Toronto: 57%; Norfolk: 57%; Vancouver: 52%; Health: individual with heart or lung disease in the household: New Brunswick =38%; Toronto =37%; Norfolk =38%; Vancouver =30%.
Sugerman et al. [31]	JA	CSS	San Diego May-Jun 2008	*N* = 1802	Community of San Diego present during wildfire	Age (group) (*n* = 1689 considered): 18–34: 15.8% (*n* = 266); 35–54: 46.5% (*n* = 785); ≥55–64: 36.8% (*n* = 638); Gender: female: 49.6% (*n* = 855), (*n* = 1724 considered). Ethnicity: non-Hispanic White: 65.3% (*n* = 1090); Hispanic: 22.1% (*n* = 369); Asian: 5% (*n* = 84); Non-Hispanic Black: 4.1% (*n* = 68); Other: 3.5% (n = 58). Health: asthmatics: 15.1% (*n* = 272); COPD/emphysema: 2.7% (*n* = 48); Heart failure: 2.9% (n = 53).
Wen et al. [32]	JA	CSS	Colorado, Florida, Indiana, Kansas, Massachusetts, & Wisconsin 2005	N = 33,888: for the relevant questions response rate ranged from n = 13,979 to *n* = 28,276.	Residents of six US states	Health (N = 28,693 considered): self-reported asthma (*n* = 4295). Other demographics were not reported for the overall sample, but only for people who changed behaviour.

*CP* Conference Proceeding, *CSS* Cross-sectional survey, *ICS* Inhaled corticosteroid, *ICS+ LABA* Combination therapy (ICS + Long-acting beta agonist), *JA* Journal article, *Pre-Post T* One-group pre- post-test (no randomisation or control group), *QES R & P* Retrospective & prospective data linkage cohort study, *QES* quasi-experimental study cross-sectional study, *QS* Qualitative study, *R* Report, *T* Thesis, *WP* Working paper

Whilst a few studies focused on health warnings associated with ground-level ozone (smog) forecasts and alerts systems [15, 26, 42], the majority of the studies focused more on comprehensive and recent air quality forecasts and alerts with associated health advice [24, 25, 27–38, 40, 41, 43], one study used a web-based asthma action plan smartphone application [39], and one study investigated responses to emergency risk communications during a local wildfire episode [31]. All the studies focused on health warnings targeting to some extent either or both of the following broad categories: the generally healthy and people at greater risk of experiencing symptoms (see Additional file 3: Table S2). However, only one study [39] provided individually tailored health information, based on recipients’ clinical data. Six studies [25, 34, 40, 42, 44, 45] investigated actual adherence to messages delivered exclusively through non-personal channels such as newspapers and government websites. In these studies, average adherence rates ranged from 20% [40] to 98% [45], with median adherence 37.9%. Four studies [24, 35, 38, 39] focused on messages delivered exclusively through personal channels such as telephone messages, email notifications, and smart phone applications. In these studies, average adherence rates ranged from 39.7% [38] to 84.6% [24], with a median of 50% (Lyons et al. [36] was not considered here as they used an indirect measure of adherence based on health care utilisation). The remaining 6 studies [26, 29, 30, 32] focused on messages delivered through both personal and non-personal channels, or did not specify method of delivery [33, 43]. Additional file 3: Table S2 provides information about the type of air quality information considered for each study, the delivery format of the air quality information provided, as well as adherence rates and self-reported reasons for, and predictors of actual and intended adherence or non-adherence.

Adherence to health advice associated to air quality information was investigated via non-validated self-report questionnaires or interviews in all but one study [36], which used objective emergency department attendances, general practitioner contacts and prescribed medications data as measure of efficacy of the alerts in decreasing health services utilisation. Seventeen studies investigated actual adherence, with only three studies investigating intended adherence [27, 28, 29], and one study assessing both actual and intended behaviour [15]. Amongst the studies investigating only intended adherence, two measured reduction in outdoor activities during air pollution episodes [27, 28], whilst one investigated other types of protective behaviours as well as outdoor activity reduction [37]. Amongst the studies investigating actual adherence, eleven [15, 25, 26, 29, 30, 32–34, 40–42] focused specifically on behavioural changes such as reducing or rescheduling outdoor activities during periods of poorer air quality, whereas a total of seven studies [24, 31, 35, 36, 38, 39, 43] assessed other forms of behaviour, such as: taking their medication with them in case of need; reliever and/or preventer asthma medication use; changes in travel route and means of transport; emergency department attendance; hospital admission; GP visits; taking leave from work; keeping kids from school; closing windows and wearing masks. See Additional file 3: Table S2.

### Assessment risk of bias

The results for the assessment of the risk of bias in the relevant studies are reported in Table 3. We identified serious methodological flaws in several of the included studies. According to our assessment, only five studies had a low risk of selection bias [15, 26, 31, 33, 36], two studies had a low detection bias [36, 41], and three had a low reporting bias [32, 36, 41]. Three studies had high [24, 38, 39] risk of other sources of bias, based on the adoption of small or inadequate sample sizes. Many studies had moderate to high selection bias, with no clear definition of source of population and response rates. As a result, we have doubts about the generalizability of the results. The fact that the majority of studies did not use psychometrically validated scales to measure adherence means that we cannot be sure those questionnaires measured what they claimed to measure. This also made it difficult for us to compare results across studies. Finally, some of the most common issues associated with reporting results included failure to report confidence intervals, which provide information about a range in which the true population value lies, and account for confounders either in the design or in the analyses. The implication is that for the majority of the included studies we cannot be sure whether unaccounted confounding factors may explain their results. Given these intrinsic problems, the results of our systematic review have to be taken with caution.

**Table 3:** Assessment of risk of bias in included studies

Author	Selection bias	Detection bias	Reporting bias	Other sources of bias
Evans et al. [26]	L	M	M	L
Hartill [24]	H	M	H	H
Johnson [27]	M	M	H	L
Johnson [28]	M	M	M	L
Kentucky Health Issues Poll [34]	H	M	H	L
Kilbane-Dawe et al. [35]	H	M	H	L
Licskai et al. [39]	H	H	H	H
Lyons et al. [36]	L	L	L	L
Mak, et al. [43]	H	M	H	L
Mansfield et al. [33]	L	M	M	L
Laube [38]	M	M	M	H
McDermott et al. [25]	H	M	H	L
Radisic, et al. [41] (qualitative)	M	L	L	L
Radisic et al. [40]	M	M	H	L
Reams et al. [29]	H	H	H	M
Semenza et al. [30]	M	M	H	L
Skov et al. [15]	L	M	H	L
Smallbone [37]	H	M	H	L
Stieb et al. [42]	M	H	H	L
Sugerman et al. [31]	L	M	M	L
Wen et al. [32]	M	M	L	L

*H* high risk of bias, *M* moderate risk of bias, *L* low risk of bias, *N/A* not applicable

### Adherence rates

Additional file 3: Table S2 presents separate results for studies investigating only intended behaviour change, actual behaviour change such as reduction or rescheduling of outdoor activities, and other forms of protective behaviour. When reported by individual studies, Additional file 3: Table S2 shows separately prevalence of adherence for people with and without respiratory conditions.

In the studies investigating actual adherence to the health advice to avoid, reduce or reschedule outdoor activities during poor air quality, overall adherence rates ranged from 9.7% [30] to 57% [33], with median adherence rates of 31% (Additional file 3: Table S2). However, these figures have to be treated with caution as they include also behaviour changes performed less than monthly, and different population samples and subgroups within them, which contribute to such a wide range of adherence rates. For instance, in the studies specifically comparing adherence rates in subgroups of healthy and vulnerable respondents [15, 25, 32, 40, 42], average adherence rates for healthy participants ranged from 13% [15] to 42% [25], whilst for the vulnerable subgroups ranged from 21% [40] to 70.7% for a subgroup of respondents with severe lung disease [15].

In the studies investigating a wider range of actual protective behaviours, going beyond the decision to simply reduce or reschedule outdoor activities, overall self-reported adherence (including behaviours performed less than monthly during moderate or high pollution episodes) ranged from 17.7% [43] to 98.1% [31], with median adherence rates of 46% (Lyons et al. [36] was not considered here as they used an indirect measure of adherence based on health care utilisation). The most common responses reported by all study participants included: avoiding busy or polluted road (with adherence rates ranging from about 10% to 52.5% [24, 35, 38]), spending more time indoors (ranging from about 30% to 58.7% [31, 35, 38]), adjusting or rescheduling travel or other outdoor activities (41.4%), changing means of travel (38.6%) [38], and avoiding strenuous exercise or other outdoor activities (ranging from 17.4% to 88.4% [24, 31, 35, 39, 43]). Other behaviours included taking a reliever medication (ranging between 30.5% and 50% [24, 35]), taking a preventative medication (30.5%–38.5% [24]), getting advice from GP (about 1% [35, 43]), and wearing a mask (6.4% to 8.1% [31, 43]). Lyons et al. [36] found that being registered to an air quality alert system for people with asthma was actually associated with a statistically significant increase in emergency admissions for respiratory conditions (IRR: 3.97; 95% CI [1.59–9.93]) and A&E attendance (IRR = 1.89; 95% CI [1.34–2.68]), compared to a control group of asthmatics not receiving the alerts. These results were contrary to the researchers’ expectation that receiving air alerts (which were advising people, depending on the level of air quality, to consider reducing or reduce outdoor physical activity and to follow their doctor’s usual advice in managing their condition) would reduce health service utilization, probably through increasing control of their respiratory symptoms. On the other hand, Licskai et al. [39] found that amongst a small sample of twenty-two asthmatic users of a semi-tailored asthma action plan smartphone application (SPA), there was a reduction in the total number of urgent health care visits (although pre-post- tests did not reach statistical significance). Moreover, 86% of them reported following the action plan recommendations to improve control of their asthma, although only 50% reduced or rescheduled strenuous outdoor activity at least once due to air quality notifications.

Three studies [15, 28, 37] investigated adherence intention rates in relation to hypothetical air pollution scenarios, presenting participants with above-standard air quality levels, whilst one study [27] considered both above and below standard air quality levels and reported aggregate intention rates. These studies showed an overall intention to adhere ranging from 36.4% [37] to 53% [27, 28], Median = 48.5%.

### Predictors of adherence and non-adherence

Predictors of actual and intended adherence are presented separately, as well as qualitative data on self-reported reasons for adherence and non-adherence. Given that we did not find major qualitative differences between predictors, results for predictors of adherent behaviours related to reducing or rescheduling outdoor activities and predictors of other protective behaviours are presented together.

Attempts to identify socio-demographic predictors of actual adherence to air quality alerts gave mixed results. Gender was identified as a significant predictor of adherence in five studies [15, 31–33, 40] (out of eight), where female participants were more adherent than males. Older age was also found to predict adherence in three studies (out of eight), where those aged between 45 and 54 years [40], those aged 60 years or older [43], and older participants (not otherwise specified) [38] were more adherent than younger participants. Moreover, being white was associated with lower rates of adherence in one study [33] (out of two); whereas another study found that speaking English as primary language [31], and reporting a higher level of education were positively associated with adherence [31] (out of three studies). Whilst employment status did not predict behaviour change [15, 31, 33], higher income was an inconsistent predictor of adherence, with two studies [31, 33] finding higher income positively and negatively associated with adherence respectively, and one study finding no association between the two variables [29]. Moreover, geographic factors predicted behaviour change in Radisic et al. [40], where area of residency predicted the likelihood of following health messages accompanying air quality indices. Although it is not clear how residency affected adherence, the researchers hypothesised that higher adherence rates were due to the presence of different environmental and health promoting initiatives in those areas [40]. Three studies [25, 26, 31] found that people with respiratory impairment (such as asthma or COPD) were significantly more adherent than healthy participants. However, in one of these studies, when parents were asked to specify how many times they restricted the outdoor activities of their asthmatic children using quantitative descriptors (i.e. exact frequency of behaviour) rather than qualitative descriptors (i.e. qualifiers such as ‘sometimes’ or ‘most of the time’), the differences between the asthma and non-asthma cohort disappeared [25]. Significantly higher rates of adherence in people with pre-existent health conditions were found in two studies (out of four): in particular, Wen et al. [32] found that having a disability (defined as any health problem or impairment, not limited to asthma) was a significant predictor of adherence, whereas Laube [38] found that having health problems predicted changes specifically in travel time and route during pollution episodes. Similarly, Stieb et al. [42] found that individuals with cardio-respiratory conditions were twice as likely as healthy individuals to change their behaviour due to air quality warnings (unfortunately exact figures are not reported). Differences in behaviour change between chronically ill individuals and healthy individuals were also found in a governmental health report in Kentucky, where 43.7% of those reporting to have a chronic disease (not otherwise specified) did not change their behaviour at all compared to 52.9% of the healthy respondents [34]. The only study assessing the relationship between low mood and adherence, found that being adherent to most or all of the risk communications heard during a local wildfire was significantly associated with ‘feeling depressed or apathy’ during the same period [31].

The association between prior exposure to air pollution and adherence was inconsistent. In particular, exposure to visible air pollution due to smog or smoke from fires was associated with higher adherence in only two studies out of four [31, 33]. Moreover, exposure did not predict adherence in Mak and colleagues’ study [43], which compared members of the general public with those who spend most of their work time outdoors in busy streets, but found no differences in self-reported adherence rates. Beliefs that local levels of air quality were generally poor did not predict adherence [15, 29]; however, one study found that these beliefs were positively associated with adherence in the subgroup of people with lung disease and ‘other’ employment status [15]. On the other hand, experiencing symptoms that the person ascribes to air pollution as well as higher frequency of symptoms were significantly associated with reporting adherent behaviours [15, 38]. For instance, higher frequency of symptoms predicted higher rates of protective behaviours, including changes in frequency of going out or planning activities outdoors, as well as changes in travel time and route, and changes in choice of means of transport [38]. Moreover, beliefs that something can be done to reduce local smog [26] predicted adherence, together with beliefs that air pollution can have a negative health impact [26, 38] (out of three studies), with one study specifically showing a significant association between this latter belief and changes in means of transport [38]. Personal concern seemed also to play a partial role in people’s decision to change behaviour in response to air quality alerts [34], where 41% of the ‘very concerned’ reported to have changed behaviour ‘a lot’, compared to the 29.6% of those ‘somewhat concerned’ and 9% of those ‘not at all concerned’.

Whilst knowledge about the causes of smog or pollution was not associated with adherence [15, 26], knowledge about the air quality index, and in particular understanding what it means and knowing where to find it were factors associated with higher adherence rates [40]. In line with this, other predictors of adherence included awareness of the existence of media alerts [32], and higher frequency of checking air quality information [29]. In addition, Laube (2012) [38] found that the use of an air quality smartphone app did not predict changes in going out, but only partially predicted changes in travel time and route, and in choice of means of transport; whilst use of other sources of information about air quality predicted all of the three types of behaviour changes considered. Finally, seeing a doctor [31] or receiving the advice from a health care professional to reduce outdoor activities were significantly associated with higher adherence to air quality warnings [32].

Amongst the six studies collecting data on self-reported reasons for non-adherence, four found that individuals were often relying more on their subjective perception of poor air quality rather than official air quality information to take protective actions [24, 30, 40, 41]. On the other hand, another study found that people’s behaviour was partially driven by media alerts alone (for 31.1% of those with and 16.1% of those without asthma), by individual perception of bad air quality alone (for 25.6% of those with and 12% of those without asthma), and by a mixture of both information sources (for 75.2% of those with and 68% of those without asthma) [32]. The most common self-reported reasons for non-adherence included time constraints [40, 41] and the pressure of continuing everyday life [24], lack of knowledge about where to check the health messages [40], confusion between air quality indices and other indices (e.g. hot weather) [41], and difficulties in understanding some messages (e.g. ‘What does away from busy roads mean?’) [24]. Lack of self-efficacy in checking and following health messages was also reported as a barrier of adherence [40]. Radisic et al. (2016b) found that those living in a lower economic area indicated that they did not check and did not follow the health messages since they ‘cannot change it [the situation]’, whereas those in higher economic areas indicated that checking and following AQHI health messages was ‘not a high priority’ [40]. Similarly, other studies identified amongst the self-reported reasons for non-adherence the belief that there was nothing people could do personally to change the situation [42], and the lack of personal relevance of the messages [41], e.g. ‘it does not affect me’ [42]. Study participants were also asked to indicate which factors they thought were facilitating their adherent behaviours. Amongst these factors, respondents reported beliefs about the benefits of following the health advice accompanying air quality indices [24, 40, 41], including the benefit of protecting one’s own health and the health of those cared for via familial and/or occupational duties [40, 41], and the utility of the alerts in assisting in the management of respiratory symptoms [24]. Other reported facilitators of adherence included being prompted to use the air quality indices by a health care professional, receiving air quality information that focused on a neighbourhood scale, and using wearable devices to access air quality indices [41].

Among the studies assessing intended behaviour, only one study [27] attempted to identify demographic factors as potential predictors of adherence. This study found that being a woman (both white and non-white) compared to the group of non-white men, and being an English speaker at home was associated with higher intention to limit outdoor activities during air pollution episodes [27]. However, it should be noted that when the analyses shifted from all respondents to the two subgroups of respondents exposed to air quality indices related to ‘good’ levels alone or ‘unhealthy’ levels alone, being an English speaker at home was no longer statistically significant (Additional file 3: Table S2). One US study [28] investigated the effect of different information formats (see Additional file 3: Table S2 for details about these formats). They found that reading an old air quality index format, which presented only one general ‘Unhealthful category’ (index value: 100–200), was associated with higher intention to adhere with the recommendation to limit outdoor activities - when compared to a new format separating the original ‘Unhealthful’ category into two different descriptors and index values: Unhealthy for Sensitive Groups (value: 101–150), and Generally Unhealthy (value: 151–200) -. These results appear to be meaningful when we consider that the format moderated level of concern about air pollution, with the new format reducing concern (50% of the Old format readers vs. 39% of the new format (Zadj = 3.62, *p* < 0001), and perceived sensitivity to air pollution (82% of Old readers vs. 63% of New readers agreed with ‘I am sensitive to air pollution’; Zadj = 3.98, *p* < 0.0001). Also, contrary to what expected, participants reading the format without ‘cautionary statements’ (e.g.: ‘Sensitive children and adults […], should limit prolonged, moderate exertion outdoors’, see Additional file 3: Table S2) reported higher adherence intentions. Smallbone (2010) [36] found that more people in the sensitive group (57%) intended to change behaviour compared to the non-sensitive group (21%). In addition, amongst the sensitive group, only 4% of people compared to the 21% of people in the healthy group would not alter their plans, as the information was not important to them personally. Unfortunately, the researchers did not carry out any analysis of association between adherence rates and possible predictors.

## Discussion

To the best of our knowledge, this is the first systematic review investigating predictors of, and reasons for, adherence and non-adherence to health advice aiming at encouraging the general public to reduce their exposure during air pollution episodes. Our rigorous inclusion criteria ensured that only those studies involving participants who were actually using or were aware of air quality warning systems were included. This decision has limited the number of studies included in the review, but we believe it has increased the validity of our results. Overall, we often found suboptimal adherence rates, with actual adherence to the recommendation to avoid, reduce or reschedule outdoor activities (aiming at self-protection), ranging from 9.7% [30] to 57% [33], with a median adherence rate of 31%. Other common self-reported protective behaviours included avoiding busy or polluted roads, adjusting or rescheduling travel or other outdoor activities, avoiding strenuous exercise, and taking reliever or preventative medication. Respondents also reported behaviours such as getting advice from their GP, wearing masks, accessing emergency services for respiratory conditions, keeping children at home from school, and following personal action plans aimed at improving control of their asthma. In the studies investigating this wider range of actual protective behaviours, overall adherence ranged from 17.7% [43] to 98.1% [31], with a median adherence rate of 46%. The fact that the included studies assessed very different target behaviours, used diverse and non-validated measures of adherence, as well as different samples and subgroups within them, contributes to such a wide range of rates, and makes it difficult to compare the results. Moreover, the percentages reported above have to be considered with caution for a number of reasons. First of all, they may represent an optimistic estimate of adherence, as they often refer to behavioural changes performed less than monthly or adherence to at least some of the health advice received. Secondly, there is a lack of definition of what constitutes ‘reasonable’ or ‘adequate’ adherence [25, 46]. Finally, although patients’ self-reports can simply and effectively measure adherence [46–48], self-report measures may result in higher estimates of adherence compared to objective measures [49, 50]. The difficulty in assessing the health impact of air quality warning systems is also potentially complicated by the nature of these alerts, as their aim is to promote change in multiple behaviours. These can include actions taken to reduce pollution, action taken to reduce exposure to pollution [15], and actions taken to become more aware and engaged in air pollution issues [5]. In addition, the health recommendation provided in association with air quality alerts is rarely specific and exhaustive which, in turn, can make it difficult to assess the effectiveness of these communications. Bearing this in mind, the results of our systematic review can help our understanding of what factors are associated with adherence and non-adherence. As there were no major qualitative differences between predictors, the results for predictors of different types of actual adherent behaviours or intended adherence are discussed together. Overall, attempts to identify demographic predictors of adherence to air quality alerts have given mixed results. More consistent results were associated with self-reported respiratory impairments (such as asthma or COPD) or other pre-existing health conditions, which were found to be significantly associated with adherence in five studies [25, 26, 31, 32, 38] (out of seven). However, in one [25] of these studies when participants were asked to quantify exactly the number of health messages they adhered to, rather than just using qualitative descriptors (such as ‘most of the time’), the differences between the asthma and non-asthma cohort disappeared. Although it is reassuring that more vulnerable people seem to take more protective actions against air pollution compared to healthy individuals, it is still quite worrying that adherence rates in the respiratory condition subgroup were often suboptimal, ranging from 21% [40] to 70.7% [15]. While it has been previously reported that people with health conditions and direct exposure to health threats may perceive a greater personal risk and therefore be more likely to adhere [51], our results confirm the established literature documenting the problem of non-adherence in patients with chronic health conditions (e.g. [52]), and in particular in relation to the adoption of protective behaviour during poor air quality episodes [53]. Similar to previous reviews [20], we are discussing our results using the COM-B model of behaviour change [9–11] (see Table 4). In relation to ‘psychological capability’, awareness of air quality alerts [32], knowing where to check air quality indices, and understanding what these indices mean were significantly associated with higher adherence [40]. These latter results are in line with previous research showing that in order to understand risk and to make appropriate health decisions, health literacy (including numeracy) is critical [54, 55]. It is also worth noting that some experts have raised doubts about the accessibility and readability of existing air quality information aiming at the general public [4, 56, 57].

**Table 4:** Factors influencing adherence to health advice provided in association with air quality information services (demographic factors not included)

CAPABILITY	MOTIVATION	OPPORTUNITY
*Psychological*	*Reflective*	*Physical*
• Knowing where to check AQHI (Air Quality Health Index) numbers [40] • Understanding the air quality indices/health messages [24, 40, 41] • Confusion between different indices [41] • Awareness of media alerts [32] • Use of different sources of information [38] • Information seeking behaviour [29]	• Health messages able to reduce both concern about, and perceived susceptibility to, air pollution [28] • Experiencing symptoms ascribed to air pollution (beliefs about the illness & threat) [15, 38] • Beliefs that smog can have negative health effects (beliefs about the health threat) [26, 38] • Beliefs that something can be done to reduce smog (outcome expectancies) [26] • Perceived benefits of AQI (Air Quality Index) adoption (beliefs about the recommendation) [24, 40, 41] • Perception of lack of necessity of AQI adoption (beliefs about the recommendation) [40, 42] • Lack of message relevance [41, 42] • Self-efficacy/locus of control [40, 42]	• Wearable device option/smartphone applications [36, 38, 41] • Exposure to visible air pollution [31, 33] • Pressure to continue with everyday life/Lack of time [24, 40, 41]
*Physical*	*Automatic*	*Social*
	• Depression [31] • Reliance on sensory cues [24, 30, 40, 41]	• Professional health care network promotion/GP advice [31, 32, 41] • Neighbourhood scale focus [41] • Local media reporting [40, 41]

Another factor associated with higher adherence was information-seeking behaviour about air quality [29]. This factor was less easy to categorise within the COM-B, as it could be classified as both part of ‘psychological capability’ and ‘reflective motivation’. On the other hand, amongst the factors related to ‘automatic motivation’, experiencing depression or apathy, as self-reported, during a local wildfire episode was found to be associated with being adherent to most or all of the risk communications received during this episode [31]. This result is quite surprising as previous meta-analyses have highlighted the opposite phenomenon, where depression works as a barrier to adherence to health advice [58]. However, we have to acknowledge two aspects here: a) the measure of low mood seems to refer only to the limited period of the fires and not to a chronic condition; b) it is not clear whether mood has been assessed using standardised measures. Another factor related to ‘automatic motivation’ was people’s reliance on sensory cues to detect air quality. In particular, four studies [24, 30, 40, 41] (out of six) found that many respondents were reporting to have reduced or rescheduled outdoor activities based on their own perception of low air quality rather than in response to air quality alerts on the same day. We know from previous studies that the most common way to detect air pollution is via subjectively experienced health effects (e.g. symptoms) and sensory cues (e.g. visual or olfactory) [4, 37, 59–63]. Although a positive correlation between people’s own perception of air quality and official monitoring data has often been reported [28, 62, 64–66], other studies did not find this correlation [30, 67]. This means that our senses may not always provide us with accurate information about air pollution [68] and this could constitute a barrier to adherence to air quality warnings. On the other hand, as Johnson [7] has argued, both official data and sensory data may be ‘accurate’ even though they may not necessarily provide identical cues for air pollution. For instance, one of the studies included in this review has demonstrated that air alerts and personal perceptions could work together to maximise chances of adopting protective behaviours. They found that 31% of those with and 16% of those without asthma changed behaviour in response to media alerts alone, whereas 26% of those with and 12% of those without asthma did so because of their individual perception of bad air quality alone, and a total of 75% of those with and 68% of those without asthma did so using both information sources [32]. To maximise adoption of appropriate protective behaviours amongst the public, future research should explore how we can integrate the immediate information we receive via our senses with official information, to improve individuals’ perceived salience of the air quality indices and advice.

In addition, we identified several factors influencing adherence that related to the ‘reflective motivation’ component of the COM-B model. These included beliefs that subjectively experienced symptoms were caused by air pollution [38], and beliefs that air pollution can have a negative health impact, also referred to as perceived severity, [26, 38]. Moreover, amongst the barriers to adherence, we identified factors such as being exposed to health messages perceived to be not personally relevant [41, 42], as well as to health messages able to reduce both concern about air pollution and perceived susceptibility to air pollution [28]. These findings support the argument that when the general public does not perceive air pollution as a ‘personal’ risk, with a direct potential short term effect on health, it is less likely they will change their behaviours [51, 69, 70]. The importance of risk appraisal, including both cognitive and emotional factors, is also confirmed by a meta-analysis conducted by Sheeran et al. [71]. In this, they showed that when behavioural change interventions are successful in heightening people’s perceived severity and perceived susceptibility to the threat (if no preventative action is taken), factors such as worry or concern about the threat and anticipated feelings of regret influence health-related behaviours. Importantly, they also found that the effects appraisal were augmented when self-efficacy (i.e. beliefs about one’s ability to perform the recommended behaviour), and response efficacy (i.e. beliefs about the efficacy of the health advice received) were stronger, and when perceived response cost (i.e. beliefs about the negative consequences associated with the recommended behaviour) were lower. For instance, it has been emphasised by several researchers [72–76] that, to be effective, fear or worry generating approaches also need to promote higher perception of efficacy, through providing specific advice on how to manage the health risk presented. This evidence is consistent with the factors identified in this review as facilitating adherence, such as beliefs about the efficacy of the health advice in protecting the health of both the individual and their family [40, 41], together with outcome expectancies and, in particular, beliefs that something can be done to reduce air pollution [26]. Moreover, amongst the barriers to adherence, we identified lack of self-efficacy and lack of internal locus of control, perceived non-necessity to follow the health advice [40, 42], perceived lack of time to check and follow health advice [24, 40, 41]. This latter result is consistent with the argument that preventative behaviours that interfere with other daily activities are less likely to be performed [77].

Other identified facilitators of adherence included receiving advice from a health care professional about reducing exposure to air pollution [32] (categorised in the ‘social opportunity’ component of the COM-B), and being able to access a wearable device providing information about air quality (categorised as ‘physical opportunity’) [41]. For instance, one study [38] found that although the use of an air quality smartphone app did not predict changes in going out, it partially predicted changes in travel time and route, and in choice of means of transport. Moreover, another study [39] found that amongst a small sample of asthmatic users of a semi-tailored asthma action plan smartphone application, there was a reduction in the total number of urgent health care visits (although pre-post- tests did not reach statistical significance), and relatively high rates of adoption of their personalised asthma action plan (including use of asthma control medications), although only 50% reduced or rescheduled strenuous outdoor activity at least once due to air quality notifications. It is worth noting that although the asthma action plan was highly customised to the message recipients, the advice to reduce exposure to air pollution was not. This aspect may partially explain these results [78, 79]. On the other hand, another study [36] found that individuals with asthma who were registered to a non-tailored air quality alert system, compared to a control group of asthmatics not receiving the alerts, accessed emergency services for respiratory symptoms significantly more. These results were contrary to the researchers’ expectation that receiving air alerts would reduce health service utilization, probably through reducing exposure to air pollution and consequently reducing chances of a respiratory crisis. The researchers tried to explain their results as being due to an increase of awareness and worry, which led to an inappropriate use of emergency health care utilization. However, this is somewhat speculative as they did not measure perceived worry or other predicting variables. Moreover, we tried to understand whether actual adherence rates differed depending on whether the health warnings were received through personal or non-personal channels. However, the data available did not allow us to draw any conclusions. In general, these results are consistent with previous studies recognising wearable devices as possible facilitators, and not necessarily drivers, of health behaviours [80], where engagement strategies [17] and message tailoring [78, 79] may be the key to successful health messages, particularly when these messages address people’s beliefs about the health threat and the advice [81]. Finally, it is worth noting that whilst a growing body of research is showing that air quality warnings have the potential to reduce hospitalisation due to respiratory symptoms, as well as reduce to some extent outdoor physical activities during air alert days [7, 53, 82–93], we cannot be entirely sure that these observed behaviours are primarily driven by the warnings themselves rather than people’s own perception of bad air quality or perhaps a combination of the two. This is because these studies are mainly based on the assumption that everyone is exposed to air quality alerts when these are issued.

This review has many limitations. We have searched only for articles published in English. Moreover, we cannot exclude the possibility of publication bias where published studies might have systematically different results compared to unpublished studies. However, to mitigate this problem we have also conducted a grey literature search. In addition, only the lead researcher (DD) manually screened all the articles and performed data extraction, although three co-authors (DD, VA and LS) independently validated the final list of included studies, and three co-authors (DD, JW and LS) independently validated the assessment of risk of bias for the included studies. We also identified serious methodological flaws in several of the studies, including presence of risk of selection bias, detection bias and reporting bias in many studies, as well as the use of small or inadequate sample sizes in some studies. Common flaws included poor reporting of response rates, limited consideration of confounders either in the design or analyses, and poor reporting of confidence intervals and effect sizes. In addition, the majority of studies used non-validated measures of adherence, and only a minority of studies compared adherence rates in subgroups of healthy and vulnerable respondents. Furthermore, the reviewed studies used different definitions of adherence, focused on diverse air quality warning systems and associated health advice, and investigated different outcome behaviours (e.g. from staying indoors to changing travel routes, wearing masks, or accessing emergency care) and samples. It is also worth noting that this review included studies covering a long period of time (from 1982 to 2016), which means that we compared messages that were referring to different types of measures of air pollution (from smog forecasts to more complex measurement of different particulate matters). Moreover, these messages were delivered using different technologies, moving from more general information on newspapers to more dynamic and real-time information provided via smart-phone apps. Throughout the years people’s perceptions of air quality might have also changed a lot, together with different attitudes towards air quality information and protective behaviours. Therefore, our aggregate results must be considered within such a heterogeneous group of studies. Although the presence of these factors has broadened the results, at times this made comparisons between studies difficult.

## Conclusions

The present review found frequent suboptimal levels of adherence to health advice accompanying air quality alerts and indices. It has also identified several facilitators of and barriers to adherence. Although demographic factors did not consistently predict adherence, several psychosocial facilitators of adherence were identified. These include knowledge on where to check air quality indices, beliefs that subjectively experienced symptoms were due to air pollution, perceived severity of air pollution, and receiving advice from health care professionals. Barriers to adherence included: lack of understanding of the indices, being exposed to health messages that reduced both concern about air pollution and perceived susceptibility, as well as perceived lack of self-efficacy/locus of control, reliance on sensory cues and lack of time. The psychosocial factors influencing adherence and non-adherence identified in this review can be used to inform public health communications used during air pollution episodes and aimed at enabling the general public to adopt protective behaviours.

## Additional files

Additional file 1: Full search strategy. This file provides the complete list of search terms used to search MEDLINE, EMBASE, PsycINFO, Science Direct, CINAHL, and other databases. (DOCX 18 kb)

Additional file 2: Criteria for assessment of risk of bias. This file contains the tool used to assess the risk of bias for the studies included in the review. The tool was adapted from the Scottish Intercollegiate Guidelines Network (SIGN) critical appraisal methodology checklist for cohort studies, and supplemented by relevant items from the Cochrane Collaboration’s Risk of Bias tool. (DOCX 41 kb)

Additional file 3: Data extraction showing the main results of the included studies. This file contains the data extraction results for all 21 articles included in this review. Data included authors, type of air quality information and type of health advice considered, information delivery format (including details on message provider, target population, channel used, and whether the message was tailored or not), measure of adherence, adherence rates, self-reported reasons for, and predictors of adherence and/or non-adherence. (DOCX 102 kb)

## Abbreviations

AQHI: Air Quality Health Index
AQI: Air quality index
CO: Carbon monoxide
COM-B: Capability, Opportunity, Motivation framework
COPD: Chronic obstructive pulmonary disease
CSS: Cross-sectional survey
DEFRA: Department for Environment Food & Rural Affairs
ICS: Inhaled corticosteroid
ICS+ LABA: Combination therapy (ICS + Long-acting beta agonist)
JA: Journal article
NIHR HPRU: National Institute for Health Research Health Protection Research Unit
NO_2_: nitrogen dioxide
O_3_: Ozone
PHE: Public Health England
PM_2.5_ and PM_10_: fine particulate matter
Pre-Post T: One-group pre- post-test
PRISMA: Preferred Reporting Items for Systematic Reviews and Meta-Analyses
QES: Quasi-experimental study cross-sectional study
QES, R & P: Retrospective & prospective data linkage cohort study
QS: Qualitative study
R: Report
SIGN: Scottish Intercollegiate Guidelines Network
SO_2_: Sulphur dioxide
T: Thesis
TRI: Toxic Release Inventory
TRS: Total reduced sulphur
WHO: World Health Organisation
WP: Working paper
