# Supplementary material for: Psychosocial and demographic predictors of adherence and non-adherence to health advice accompanying air quality warning systems: a systematic review
Source: Environ Health. 2017 Sep 22;16:100. doi: 10.1186/s12940-017-0307-4 (PMC5610416; doi:10.1186/s12940-017-0307-4)
Supplement: Supplementary file 1 — Full search strategy. This file provides the complete list of search terms used to search MEDLINE, EMBASE, PsycINFO, Science Direct, CINAHL, and other databases. (DOCX 18 kb) [file 12940_2017_307_MOESM1_ESM.docx]

**Additional file 1: Full search strategy**

**OVID (Global Health), PsycINFO, Social Policy and Practice, Embase, Ovid MEDLINE(R)**

1 or 2 or 3 or 4 or 5 or 6 or 7 or 8 or 9 or 10 or 11 or 12 or 13 or 14 or 16 or 17 or 18 or 19 or 20

("air quality alert*" or "air quality advi*" or “air quality index" or "air quality indices" or "pollution forecast*" or air pollution communicat* or air quality communicat* or air pollution messag* or air quality messag* or "air pollution advi*" or air quality inform* or "media alert*" or "air pollution alert*" or air quality forecast* or environmental health. or "ozone alert*" .or PM 25 or smog advisor* or smog alert* or mobile-based intervention*)

AND

( exp compliance/ or adherence or medication adherence or (compliance or compliant) or (adherence or adherent) or public response* or intention* or (public or population) or response* or reaction* or decision* or behavio?ral impact .or outdoor activit* or health behavio?r* or behavio?r* change* or behavio?r modification* or behavio?r* response* or (protective behavio?r* or "protective measure*") or self-protection or avoidance behavio?r or risk reduction or transport* or (efficacy or effective or impact)

OR

asthma* or inhaler or COPD or at risk population

**Limit: English language**

**Science Direct**

tak(**compliance OR adherence)** OR compliant OR adherent OR {medication use} **OR {public response*} OR intention* OR {protective measure*}** OR response* OR public OR respond* OR decision* OR choice* OR {outdoor activit*} OR {health behavio?r} OR behavio?r change* OR {protective behavio?r} OR protective OR protection OR avoidan* behavio?r OR population OR transport OR {risk reduc*} **OR efficacy OR effective OR impact** **OR asthma OR COPD OR {risk population*}** OR inhaler) **AND ({pollution forecast*}** OR {air pollution} communicat* OR {air quality} communicat* OR {pollution advi*} OR {air quality} inform* OR {air pollution alert*} OR {air quality forecast*} OR {mobile-based intervention*} OR {air quality alert*} **OR {air quality advi*} OR {air quality index} OR {air quality indices}** OR {ozone alert*} OR smog advi* OR {smog alert*} OR {pollution warning*} OR {media alert*}).

**Limit: English language**

**Scopus**

TITLE-ABS-KEY ( "air quality alert*"  OR  "air pollution alert*"  OR  "air quality advi*"  OR  "smog alert*"  OR  "pollution forecast"  OR  "air quality ind*"  OR  "air quality warning*"  OR  "media alert*" )  AND  ( "intention*"  OR  "protective measure*"  OR  "response*"  OR  "respond*"  OR  "chang*"  OR  "complian*"  OR  behavio?r  OR  "avoidance"  OR  "protective"  OR  "outdoor activit*"  OR  "transport*"  OR  public  OR  choice  OR  "medication use"  OR  adheren* ) AND NOT  ( indoor )

**Limit: English language**

**Pubmed**

Search ((((("pollution forecast*" OR "pollution communicat*" OR "pollution advi*" OR "air pollution alert*" OR air quality forecast* OR "air quality alert*" OR "air quality advi*" OR "air quality index" OR "air quality indices" OR "ozone alert*" OR smog advi* OR smog alert* OR pollution warning* OR "media alert*"))) AND ((compliance OR adherence OR compliant OR adherent OR "protective measure*" OR prevent* OR "medication use" OR "public response*" OR response* OR public OR respond* OR decision* OR choice* OR "outdoor activit*" OR "health behavior" OR "health behaviour" OR "behaviour* change*" OR "protective behavior" OR "protective behaviour" OR avoidan* OR intention* OR population OR transport OR risk reduc* OR efficacy OR effective OR impact OR asthma OR COPD OR "risk population" OR inhaler) NOT indoor NOT water NOT smoking)))

**Limit: English language**

**CINAHL**

TX(compliance OR adherence OR medica* adherence OR compliant OR adherent OR intention* OR medication use OR public response* OR response* OR public OR respond* OR decision* OR choice* OR outdoor activit* OR health behavior OR health behaviour OR behaviour change* OR protective behavior OR protective behaviour OR avoidan* OR population OR transport OR "risk reduc*" OR efficacy OR effective OR impact OR asthma OR COPD OR "at risk" OR inhaler) AND (pollution forecast* OR air pollution communicat* OR air quality communicat* OR "pollution advi*" OR air quality inform* OR "air pollution alert*" OR air quality forecast* OR "mobile-based intervention*" OR "air quality alert*" OR "air quality advi*" OR "air quality index" OR "air quality indices" OR "ozone alert*" OR smog advi* OR smog alert* OR pollution warning* OR media alert*)

**Limit: English language**

**Web of Science Core Collection**

# 7 3,317

(#6 AND #4) AND LANGUAGE: (English) AND DOCUMENT TYPES: (Article OR Abstract of Published Item OR Proceedings Paper OR Review)

Indexes=SCI-EXPANDED, SSCI, CPCI-S, CPCI-SSH, ESCI Timespan=All years

# 6 9,973,515

#5 OR #3

Indexes=SCI-EXPANDED, SSCI, CPCI-S, CPCI-SSH, ESCI Timespan=All years

# 5 496,593

(TS=(asthma* OR inhaler OR COPD OR 'risk population')) AND LANGUAGE: (English) AND DOCUMENT TYPES: (Article OR Abstract of Published Item OR Proceedings Paper OR Review)

Indexes=SCI-EXPANDED, SSCI, CPCI-S, CPCI-SSH, ESCI Timespan=All years

# 4 4,750

#2 OR #1

Indexes=SCI-EXPANDED, SSCI, CPCI-S, CPCI-SSH, ESCI Timespan=All years

# 3 9,924,132

(TS=(compliance OR adherence OR "medica* adherence" OR compliant OR adherent OR "medication use" OR "public response*" OR response* OR intention* OR "protective measure*" OR public OR respond* OR decision* OR "outdoor activit*" OR "health behavio$r" OR behavio$r change* OR "protective behavio$r" OR protective OR effective OR efficacy OR impact OR outcome* OR avoidance OR "avoidant behavio$r" OR population OR transport OR exposure OR risk reduc*)) AND LANGUAGE: (English) AND DOCUMENT TYPES: (Article OR Abstract of Published Item OR Proceedings Paper OR Review)

Indexes=SCI-EXPANDED, SSCI, CPCI-S, CPCI-SSH, ESCI Timespan=All years

# 2 4,101

(TS=("pollution forecast*" OR "air pollution" communicat* OR "air pollution advi*" OR "air quality" inform* OR "air pollution alert*" OR "air quality forecast*" OR "mobile-based intervention*")) AND LANGUAGE: (English) AND DOCUMENT TYPES: (Article OR Abstract of Published Item OR Proceedings Paper OR Review)

Indexes=SCI-EXPANDED, SSCI, CPCI-S, CPCI-SSH, ESCI Timespan=All years

# 1 792

(TS=("media alert*" OR "air quality alert*" OR "air quality advi*" OR "air quality index" OR "air quality indices" OR "ozone alert*" OR "smog advi*" OR "smog alert*" OR "air quality warning*" OR "air pollution warning*" OR "air pollution episode*")) AND LANGUAGE: (English) AND DOCUMENT TYPES: (Article OR Abstract of Published Item OR Proceedings Paper OR Review)

Indexes=SCI-EXPANDED, SSCI, CPCI-S, CPCI-SSH, ESCI Timespan=All years

**EThos**

air pollution OR air quality AND public OR response OR change OR choice OR behaviour

**OpenGrey.eu**

air pollution OR air quality AND public (no results when adding: response OR change OR choice OR behaviour)

**Limit: English language**
