# Supplementary material for: Psychosocial and demographic predictors of adherence and non-adherence to health advice accompanying air quality warning systems: a systematic review
Source: Environ Health. 2017 Sep 22;16:100. doi: 10.1186/s12940-017-0307-4 (PMC5610416; doi:10.1186/s12940-017-0307-4)
Supplement: Supplementary file 2 — Criteria for assessment of risk of bias. This file contains the tool used to assess the risk of bias for the studies included in the review. The tool was adapted from the Scottish Intercollegiate Guidelines Network (SIGN) critical appraisal methodology checklist for cohort studies, and supplemented by relevant items from the Cochrane Collaboration’s Risk of Bias tool. (DOCX 41 kb) [file 12940_2017_307_MOESM2_ESM.docx]

**Additional file 2: Criteria for assessment of risk of bias**

Table 5. Criteria for assessment of risk of bias. Adapted from the Scottish Intercollegiate Guidelines Network (SIGN) critical appraisal methodology checklist for cohort studies (20), and supplemented by relevant items from the Cochrane Collaboration’s Risk of Bias tool (21).

| Bias | Components |
| --- | --- |
| Selection bias | The study indicates how many of the people asked to take part did so  A clear definition of source of population and clear eligibility criteria for selection of participants are used, to ensure the sample is representative  A comparison is made between the original population and the sample |
| Detection bias | The outcomes are clearly defined  The method of outcome assessment is valid and reliable (or at least face-validity) |
| Reporting bias | Confidence intervals have been provided  Appropriate statistical analyses have been carried out  The main potential confounders are identified and taken into account in the design and analysis |
| Other bias | A power calculation is reported. If not, sample size is small, medium or large (Small, n=30–59; medium, n=60–150; large, n=150+)  The study addresses an appropriate and clearly focused question  Other bias |
|  | |
