# Supplementary material for: Psychosocial and demographic predictors of adherence and non-adherence to health advice accompanying air quality warning systems: a systematic review
Source: Environ Health. 2017 Sep 22;16:100. doi: 10.1186/s12940-017-0307-4 (PMC5610416; doi:10.1186/s12940-017-0307-4)
Supplement: Supplementary file 3 — Data extraction showing the main results of the included studies. This file contains the data extraction results for all 21 articles included in this review. Data included authors, type of air quality information and type of health advice considered, information delivery format (including details on message provider, target population, channel used, and whether the message was tailored or not), measure of adherence, adherence rates, self-reported reasons for, and predictors of adherence and/or non-adherence. (DOCX 102 kb) [file 12940_2017_307_MOESM3_ESM.docx]

**Additional file 3: Data extraction showing the main results of the included studies**

Table 6. Data extraction showing the main results of the included studies

| Author | Type of air quality information & type of health advice /  Delivery format | Measure of adherence | Adherence/non-adherence rates | Predictors of adherence/  reasons for adherence or non-adherence/ % in subgroups |
| --- | --- | --- | --- | --- |
| **ACTUAL ADHERENCE: REDUCING OR RESCHEDULING OUTDOOR ACTIVITIES** | | | | |
| Evans et al.(26) | State of California Smog Health Advisories: in Southern California since 1982, sends notifications on the day before a projected smog episode.  *Advice:* Restriction of outdoor activity is advised during an alert. Children, the elderly, and others sensitive to the effects of smog are particularly advised to reduce outdoor activity and to stay indoors. *Provider:* State of California. *Target:* general public. *Channel:* news media, major employers notified directly. *Tailoring:* No | *Self-reported.* Reduction in outdoor activity, during high pollution periods (0 = Same behaviour to 2 = Change a lot). | Mean change in outdoor physical activities during smog alerts: M=0.80 (SD=0.86) in a range from 0-2; 48.8% reported they had not changed behaviour, 28.9% said they reduced outdoor activity a lot more; 22.4% put themselves in the middle. | **Individual beliefs that smog can have negative health effects** (R=0.38, b=0.42; standardised b=0.34, p<0.05)**; the more strongly a person agreed that something can be done to reduce smog in Los Angeles** (R=0.40, b=-0.16; standardised b=-0.14, p<0.05), **having some respiratory impairment** (R=0.42, b=0.26; standardised b=0.12, p<0.05), gender, age, ozone levels (prior week exposure), and knowledge of the causes of smog. |
| Kentucky Health Issues Poll (34) | The U.S. Environmental Protection Agency issues a daily Air Quality Index.  *Advice:* ‘Good’ (0-50): Air quality is considered satisfactory. "Moderate" (51- 100): Air quality is acceptable; however, for some pollutants there may be a moderate health concern for a very small number of people. For example, people who are unusually sensitive to ozone may experience respiratory symptoms. "Unhealthy for Sensitive Groups" (101-150): Although general public is not likely to be affected at this AQI range, people with lung disease, older adults and children are at a greater risk from exposure to ozone, whereas persons with heart and lung disease, older adults and children are at greater risk from the presence of particles in the air. "Unhealthy" (151-200): Everyone may begin to experience some adverse health effects, and members of the sensitive groups may experience more serious effects. "Very Unhealthy" (201-300): everyone may experience more serious health effects. "Hazardous" (300+): The entire population is more likely to be affected.  *Provider:* Environmental Protection Agency and Communities. *Target:* community of Kentuckians. *Channel:* webpage, other not specified. *Tailoring:* No | *Self-reported.* Change or limitation in outdoor activities. [not at all, a little, a lot, don’t know] | When an air quality alert is issued, 47.8% of Kentucky adults said they do not change or limit their activities at all. 27.9% reported that they change or limit their activities a little, 21.4% said they change their activities a lot. People who answered 'don't know (2.9%) (Sample considered for this question n=1657). | *Percentages:* 23.2% and 17.3% of men (n=793) change a little and a lot respectively; 56.8% do not change; 32.3% & 25.1% of women (n=864) change a little & a lot respectively, 39.6% do not change. Of the Very Concerned (n=262) 33.7% change a little; 41% a lot; 24.2% not at all. Of those Somewhat Concerned (n=585), 33% change a little; 29.6% a lot; 35.8% not at all. Of those Not At All Concerned (n=808) 22.3% change a little; 9% a lot; 64.3% not at all. Of those with Chronic Disease (n=902) 28.9% change a little; 25.1% a lot; 43.7% not at all vs. those without chronic disease (n=750) with 26.7% changing a little; 16.9% change a lot; 52.9% not at all. [% for ‘don’t know’ not reported here]. |
| Mansfield et al.(33) | Air Quality Index (2000). Reported in any event, ozone values that exceed 100.  *Advice:* ‘Good’ (0-50) Green: Air quality is considered satisfactory. "Moderate" Yellow (51- 100): Air quality is acceptable; however, for some pollutants there may be a moderate health concern for a very small number of people. For example, people who are unusually sensitive to ozone may experience respiratory symptoms. "Unhealthy for Sensitive Groups" (101-150) Orange: Although general public is not likely to be affected at this AQI range, people with lung disease, older adults and children are at a greater risk from exposure to ozone, whereas persons with heart and lung disease, older adults and children are at greater risk from the presence of particles in the air. "Unhealthy" Red (151-200): Everyone may begin to experience some adverse health effects, and members of the sensitive groups may experience more serious effects. "Very Unhealthy" Purple (201-300): everyone may experience more serious health effects. "Hazardous" Maroon (300+): The entire population is more likely to be affected*. Provider:* Local authorities. *Target:* general public. *Channel:* not specified. *Tailoring:* no | *Self-reported.* (To those familiar with the alerts): Q. How did you change your outdoor activities on moderate/high ozone alert days? (1) I stayed indoors all or most of those days; (2) I limited my time outdoors on all or most of those days; (3) I did not change. | [Respondents who adjusted behaviour in counties that had experienced moderate or worse ozone days in summer 2000]: On ozone alert days, 57% (n=422) reported spending less time outdoors. 44% of respondents reported making no behavioural adjustments (including limiting outdoor activities or driving less). | [N=965 for this analysis]: *Non-adherence during moderate or high ozone alert days:* **being male** (vs. female (Coeff (SE) = male –0.47, (0.09), p< 0.05); **being white** (compared to Asians when asked to stay indoors on high ozone days (white= –0.38, (0.15), p<0.05); black and other race respondents (compared to Asians); age; level of education; employment status; **high income households (> $75,000)** vs. low income households (high income= –0.28, (0.13), p<0.05); self-reported health status. *Predictor of adherence:* **the occurrence of high ozone days in the same summer of 2000** (purple2000= 0.59, (0.09), p<0.05); moderate alert days. |
| McDermott et al. (25) | “Air Quality Index” (AQI). On days when the AQI nears or exceeds standards, state agencies issue “air quality alerts” or “advisories.”  *Advice:* Children in general and children with asthma in particular are potentially sensitive to air pollution. Parents are advised to curtail children’s outdoor exertion to varying degrees depending on the predicted pollution levels and whether their children have asthma or other relevant medical problems. *Provider:* Environmental Protection Agency (EPA), and state agencies. *Target:* general public. *Channel:* Local news media and government websites. *Tailoring:* No | *Self-reported by parents.* Qualitative Q: How often parents restricted their child’s outdoor play when high air pollution levels. A quantitative Q: How many times a year they restricted their children’s outdoor play because of the advisories. | 55% (n=114/208) of parents reported restricting their children’s outdoor play “some” or “most” of the time during poor air quality ('reasonable compliance'). When asked to quantify their adherence: 7% (n=15) reported restricting play 11-30 times/year ('reasonable compliance'); 49% (n=101) 0-2 times per year; 25% (n=51) 3-5 times per year; 17% (n=36) 6-11 times, & 2% (n=5) >30 per year. Of those who reported restricting their children’s play for “some” or “most” of the advisories, 11% (n=13/114) reported restricting play 11-30 times a year. | *Qualitative Q.:* **being in the asthma cohort** (64%, n=70/110 reported restricting children’s outdoor play 'some or most of the time' vs. 45% (n=44/98) in the non-asthma cohort (p =0.007). *Quantitative Q.:* the % of parents reporting that they restricted their children’s outdoor play between 11-30 times per year ('reasonable compliance') did not differ between the of the asthma cohort [8% (n=9)] and the non-asthma cohort [6% (n=6)], p = 0.57). |
| Radisic et al. (40) | The AQHI communicates the cumulative health risks for three pollutants (O_3_, NO_2_, PM_2.5_), unlike the AQI that was based on 6 pollutants and shows the single worst pollutant.  *Advice:* [Low 1-3] At risk population: Enjoy your usual outdoor activities. General population: Ideal air quality for outdoor activities. [Moderate 4-6] At risk: Consider reducing or rescheduling strenuous activities outdoors if you are experiencing symptoms. General: No need to modify your usual outdoor activities unless you experience symptoms such as coughing and throat irritation. [High 7-10] At risk: Reduce or reschedule strenuous activities outdoors. Children and the elderly should also take it easy. General: Consider reducing or rescheduling strenuous activities outdoors if you experience symptoms such as coughing and throat irritation. [Very High >10] At risk: Avoid strenuous activities outdoors. Children and the elderly should avoid outdoor physical exertion. General: Reduce or reschedule strenuous activities outdoors, especially if you experience symptoms such as coughing and throat irritation. *Provider:* the federal government ([www.airhealth.ca](http://www.airhealth.ca)). *Target:* general public. *Channel:* television, radio, government website, newspapers. *Tailoring:* no | *Self-reported.* Adoption of the AQHI: ‘‘Do you follow AQHI Health Messages which tell you when to consider reducing or re-scheduling outdoor physical activity?’ Why or why not?” | 43% (n=303/707) of all participants reported that they follow AQHI health messages. However, 37% (n=113/303) of those who reported following AQHI health messages were not checking AQHI numbers (potentially not relying accurately to the AQHI to implement protective behaviours). Full ‘AQHI adoption’ (i.e. all of the following: being aware and checking AQHI, and following AQHI health messages): 20% (n=142/707) of the participants. Within the at-risk population (≥65 years and those with pre-existing respiratory and/or cardiovascular conditions), 79% (n=253/319) have not adopted the AQHI. | Logistic regression: **following health messages: being female** (B=0.765, OR=2.148, p<0.05), **knowing where to check AQHI numbers** (B=1.314, OR=3.723, p<0.01), **residing in the west lower area of the city** (B=1.170, OR=3.221, p<0.01); **understanding what the AQHI means** (lack of understanding: B=-0.777, OR=0.460, p<0.05). Logistic regression: **Adopting the AQHI: being 45-54 years** (B=1.778, OR=5.917, p<0.05), **understanding what AQHI means** (B=1.046, OR=2.845, p<0.05), **and knowing where to check AQHI numbers** (B=1.835, OR=6266, p<0.01). *Qualitative results:* *facilitators*: perceived benefits of AQHI adoption including protection of health for self and those cared for. *Barriers*: not knowing where to check, lack of time to check /follow AQHI advice; lack of Self-efficacy: ‘Don’t know how’ to follow. Those in the lower economic area indicated a lack of control; whilst those in the higher economic areas said that checking/following the AQHI is: ‘‘not a high priority’’. Reliance on sensory cues instead and only on news/ radio. |
| Radisic et al. (41) | [see Radisic et al. 2016, above) | *Self-reported.*  [As above] | not reported | *Barriers to AQHI adoption*: Message Relevance; Index confusion; Sensory cue precedence; Time constraints. *Facilitators*: Professional network promotion; add emphasis on Health benefit; Neighbourhood scale focus; Local media reporting; Wearable device option. |
| Reams et al. (29) | Baton Rouge's Air Quality Index (AQI) includes 6 EPA air quality standards (O_3_, PM_2.5_, NO_2_, SO_2_, CO, TRS). The AQI reports provides daily forecasts.  *Advice:* The AQI is divided into six categories which correspond to specific levels of health concerns. Green (0-50; Good) Air quality is considered satisfactory, and air pollution poses little or no risk. Yellow (51-100; Moderate) Air quality is acceptable; however, for some pollutants there may be a moderate health concern for a very small number of people who are unusually sensitive to air pollution. Orange (101-150, Unhealthy for Sensitive Groups) Members of sensitive groups may experience health effects. The general public is not likely to be affected. Red (151-200; Unhealthy) Everyone may begin to experience health effects; members of sensitive groups may experience more serious health effects. Purple (201-300; Very Unhealthy) Health alert: everyone may experience more serious health effects. Maroon (301-500; Hazardous) Health warnings of emergency conditions. The entire population is more likely to be affected. *Provider:* the Louisiana Department of Environmental Quality. *Target:* general public. *Channel:* daily AQI ratings and forecasts by internet/telephone. Also local television and radio news programs. *Tailoring:* no | *Self-reported.* Limitation of outdoor activities in response to Air Quality Index (AQI) forecasts (‘yes’ or ‘no’ answers). | 36% of respondents reported having limited their outdoor activities on days with lower air quality. | EXPOSURE: a) being resident in more industrialized communities based on the TRI; b) an higher n of facilities permitted to use toxins (t=-1.988, p=.052); c) experienced environmental emergency in the last 5 years. **ADAPTIVE CAPACITY:** a) feeling informed to respond to an emergency; b) length of residence; c) **self-reported more frequent checking (1=never to 5= always) of the AQI forecasts each week** (Cramer’s V = .521, p<.025, n=52 in this analysis); d) perceiving local air quality to be generally lower (1=excellent to 5= very poor). VULNERABILITY: income, age & education. Gender and adoption of household emergency plan in case of a hazardous event. |
| Semenza et al. (30) | Air quality advisory systems in Portland and Houston.  *Advice:* In Portland, the air pollution advisory requests voluntary change in behaviour to reduce ‘‘pollution from cars, mowers, paint, and aerosol sprays’’. Information about potential health impact of smog is also provided. Citizens are referred to their health providers to obtain specific advice. In Houston, the email only contains information about health impact and states: ‘‘Active children and adults as well as people with respiratory disease, such as asthma, should limit prolonged outdoor exertion.’’  *Provider:* the Oregon Dept. of Environmental Quality [Portland], the Texas Commission on  Environmental Quality [Houston]. *Target:* general public. *Channel:* [Portland] the media, e-mail (to those registered), and messages on electronic freeway message boards; e-mail alerts [Houston]. *Tailoring:* No | *Self-reported.* Changes in activity patterns that are a direct result of air quality conditions and/or health advisories. | People in Portland and Houston were as likely to respond that they changed their behaviour on control days (when no alerts were issued) as advisory days. On average 10.5% in Portland, and 9.7% in Houston actually changed their activities during poor air quality episodes. | *Changed behaviour:* **due to individual perception of poor air quality rather than air quality advisories** (p-value not reported, only graphically presented). During the 8 days considered (including 6 days of no alerts issued), on average about 9.4% of people in Portland had changed behaviour due to their perception alone; whilst during the two alert days, about 3% changed behaviour due to the air quality advisories. In Houston, of the 7 days considered (including 1 control day), about 8% changed behaviour due to personal cues of pollution alone; whilst during the 6 alert days, about 2% changed behaviour due to the air advisories. |
| Skov et al. (15) | Two notifications: one was a daily notification of air quality (based on Nitrogen Monoxide (NO), the other was a pollution alert issued in the event of a genuine smog episode (at the time of the study the latter one was not yet in effect). The daily communications through news media about the projected level of air pollution, were given in terms of a ‘below average’, ‘average’, or ‘above average’ message.  *Advice:* children, old people, and persons who might be sensitive to air pollution due to lung disease should use the notification to plan their daily activities, and possibly stay indoors during ‘above average’ pollution. *Provider:* City Council & Environmental Protection Agency. *Target:* general public. *Channel:* news media. *Tailoring:* No. | *Self-reported. (Both actual & intended change):* Avoidance of outdoor activities in response to daily air quality notifications (asked to those aware of the notifications), and in case of a hypothetical pollution alert issued on a work day (all). | Overall adherence: 21.5%. Avoidance of outdoor activities in response to daily air quality notifications about air quality levels: Healthy responders: 13% (n=60); Mild lung disease: 33.3% (n= I5); Moderate Lung disease: 53.5% (n=23); Severe Lung Disease: 70.7% (n=29). Amongst all respondents, who were asked about intended behaviour in case of a smog alert being issued: 38.5% (n=261) of healthy responders would avoid outdoor activities; Mild lung disease: 54.1% (n=40); Moderate Lung disease: 60.8% (n=31); Severe Lung Disease: 81.8% (n=45). | *Only actual change:* Being a respondent with ‘other’ employment status; being **female** (p=0.005, X^2^ value not reported); **having experienced symptoms ascribed to the air pollution** (p <0.001, X^2^ value not reported). Knowledge and beliefs about the air pollution (e.g. beliefs about the degree of air pollution, the sources of the air pollution, or beliefs about the possible adverse health effects); however, **among the lung diseased with ‘other’ employment status beliefs about the degree of air pollution was associated with avoiding outdoor activity** (p= 0.003, Fisher’s exact test). |
| Stieb et al. (42) | The Canadian Smog Advisory Program informs the public about smog episodes.  *Advice:* they describe sources of emission, how to reduce emissions, and what are the risks associated with smog exposure (i.e. O3), e.g.: ‘Commonly reported symptoms include irritation of the nose and throat, cough and chest tightness. Minimize your exposure by avoiding outdoor exercise particularly in the afternoon and early evening when ground-level ozone concentrations tend to be at their highest.’ ‘Children tend to be more sensitive than adults because they breathe faster and in the summer spend more time outdoors being physically active. Reduce your child's exposure by encouraging outdoor activities early in the day when pollutant levels are lower.’ *Provider:* Environment Canada, provincial and local governments. *Target:* general public. *Channel:* television or radio, and newspapers. *Tailoring:* No | *Self-reported.* Response to advisories: 'Did you or someone else in your household do anything differently as a results of hearing this smog advisory?' | In all areas, on average < 30% of responders (~ range 10-40%) reported that they or someone else in their household do anything differently as a results of hearing this smog advisory [including both pro-environmental & protective behaviour]. | **Individuals with cardiorespiratory disease were twice as like as the healthy individuals to take action (exact figures no reported).** Self-reported reasons for not taking action (using an open-ended question), the most common were: 'there was nothing I could do', 'I was unable to do anything', 'it was not necessary to do anything/ smog is not a problem', 'it does not affect me (i.e. no health problems)'. No % reported. |
| Wen et al. (32) | Media alerts on AQI.  *Advice:* Typical from Air Quality Index: alert people that they should limit outdoor activities to avoid ambient air pollution when the AQI is more than 100––especially children, the elderly, and people with cardiopulmonary diseases, such as asthma. *Provider:* Environmental Protection Agency and States. *Target:* general public. *Channel:* media, including television, radio, newspapers, broadcast telephone messages, and the Internet. *Tailoring:* No | *Self-reported.* Change in response to AQI. Respondents were classified into 2 groups: ‘no activity change’ and ‘activity change.’ In the latter was classified any respondent who reported changing or reducing outdoor activity ≥ 1 times during the past 12 months. | Overall adherence: 18.1%. 31% (95% CI: 27.8–34.4%) of the respondents with lifetime asthma (total n with asthma n = 1,869 considered for this question) and 16% (95% CI: 15.1–17.1%) of those without asthma (total n used 12,110) reported changes (≥1 in the last 12 months) in outdoor activities because of media alerts. | *Adherence:* **awareness of media alerts** (for those aware of air quality alerts with asthma (aOR = 2.16, 95% CI: 1.61, 2.90) & those aware without asthma (aOR = 1.72, 95%CI: 1.50, 1.98) vs. the unaware); **being female** (women without & with asthma respectively 20.9% (95%CI:19.7, 22.1) & 35.6 % (CI:31.7, 39.5) vs. men without & with asthma 12.0% (CI:10.6, 13.4) & 24.3% (CI:18.2, 30.4), p<0.05]; **disability** (Disability without & with asthma (20.7%, CI:18.3-23.1, & 40.7%, CI:35.0-46.4) vs. respondents with no asthma nor disability: 15.1% (CI:14.1-16.1) & 26.0% of asthmatics without disability (CI:21.9-30.1), p<0.05); age; **receiving health professionals’ advice to reduce outdoor activity** (for those without and with asthma (51%, CI:43.2–57.9%; and 57%, CI:49.1–63.7%; p<0.001)). *Reported reasons for change (%):* **individual perception of bad air quality alone** [25.6% (SE=1.2%) of those with and 12% (SE=0.3) of those without asthma]; **awareness of the media alerts on bad air quality alone** (31.1% (SE=1.7%) of those with and 16.1% (SE=0.5%) of those without asthma); **both of the above** (75.2% (SE=2.9%) of those with and 68% (SE=1.6%) of those without asthma). |
| **ACTUAL ADHERENCE: NOT ONLY REDUCING OR RESCHEDULING OUTDOOR ACTIVITIES** | | | | |
| Hartill (24) | Air Alert is a warning system that sends a voice or text message directly to a mobile, home phone or email to registered users. Forecasts for the next day are generated if air quality is forecast to be moderate or above.  *Advice:* Low (1-3): effects are unlikely to be noticed even by those who are sensitive to air pollution. Enjoy your usual outdoor activities. Moderate (4-6): sensitive people may notice mild effects but these are unlikely to need action. Adults and children with lung problems, and adults with heart problems, who experience symptoms should consider reducing strenuous physical activity, particularly outdoors. High (7-9): sensitive people may notice significant effects and may need to take action. Adults and children with lung problems and adults with heart problems should reduce strenuous physical exertion, particularly outdoors and particularly if they experience symptoms. People with asthma may find they need to use their reliever inhaler more often. Older people should also reduce physical exertion. Very High (10): effects on sensitive people may worsen. Adults and children with lung problems, adults with heart problems, and older people, should avoid strenuous physical activity. People with asthma may find they need to use their reliever inhaler more often. *Provider:* Eastleigh Borough Council & Southampton City Council. *Target:* users (with respiratory condition). *Channel:* personal via voice/text message, email. *Tailoring:* No. | *Self-reported.*  In 2011: Q. Do you modify your behaviour during air alerts? Q.: If you have answered 'Yes' or 'Sometimes', what did you do? (Multiple choice).  In 2014: Q. How often do you change your behaviour during Air Alert messages? Q. In what way do you change your behaviour? (Multiple choice). | In 2011: 37.7% (of n=74) reported that they modify their behaviour during air alerts ‘most of the time’; 46.4% said ‘Sometimes’ and 15.9% said ‘No’. Among those who replied ‘yes, most of the time/ sometimes’, 81.4% carried their asthma inhaler with them at all times; 30.5% took reliever medication; 52.5% avoided busy roads; 30.5% took a preventative dose of medicine; 39% avoided outdoor exercise; 11.9% replied ‘other’. In 2014, of the 52 respondents 5.8% reported that they ‘always’ change their behaviour during Air Alert messages; 36.5% ‘Most of the time’; 42.3% ‘Sometimes’; 7.7% ‘Rarely’; 5.8% ‘Never’; 1.9% did not answer. 65.4% said that they carry their inhaler with them at all times; 38.5% take reliever medication; 50% avoid busy roads; 38.5% take a preventative dose; 26.9% avoid outdoor exercise; 17.3% replied ‘Other’. | *Users’ free comments: Barriers:* it is difficult to avoid pollution when they receive an alert due to the pressure of continuing everyday life; perception of air quality change before receiving air quality alerts; difficulty in understanding the messages, e.g. ‘What does away from busy roads mean?’.  *Facilitators:* Professional network promotion; alerts help to manage symptoms. |
| Kilbane-Dawe et al. (35) | The Croydon airTEXT project directly broadcasted air pollution episode forecasts, based on the UK Air Quality index (2006). The forecasts were issued daily between 3pm-7pm for the following day. When maximum daily concentrations of O_3_, NO_2_ or PM_10_ were forecast to exceed the moderate thresholds an alert was issued at 7.30pm to registered users.  *Advice:* Low: Effects are unlikely to be noticed even by individuals who know they are sensitive to air pollutants; Moderate: Mild effects, unlikely to require action, may be noticed amongst sensitive individuals. High: Significant effects may be noticed by sensitive individuals and action to avoid or reduce these effects may be needed (e.g. reducing exposure by spending less time in polluted areas outdoors). Asthmatics will find that their 'reliever' inhaler is likely to reverse the effects on the lung. Very High: The effects on sensitive individuals described for 'High' levels of pollution may worsen. *Provider:* UK Committee on Medical Effects of Air Pollution & local authorities. *Target:* community. *Channel:* personal via SMS, voicemail & email alerts. *Tailoring:* No | *Self-reported.* Response to air alerts (provided within 2 days of receiving one) Possible responses: (‘I went out, but avoided busy roads’; ‘I went out, but did less strenuous exercise than usual’; ‘I spent more tie indoors than usual’; ‘I stayed indoors all day’; ‘Other’). | 42% reported doing something differently as a result of receiving an alert, including: I went out, but avoided busy roads (<10%); I went out, but did less strenuous exercise (~30%); I spent more time indoors (~30%); I stayed indoors all day (~30%). Health changes reported: Took usual dose of inhalers/ medication (30%); Took extra dose of medication (50%); I saw the GP: (~1%); I went to A&E/Walk-in Centre (0%); I was admitted to hospital (0%); Other (~3%). They collected also annual evaluation surveys (n=65): (no results reported). | Not considered |
| Laube (38) | London Air smart phone app, providing almost real-time air quality data at street level, air quality forecasts & guidelines. It uses the UK Air Quality Index (2012), newly recommended.  *Advice:* Low 1–3 [at risk]: Enjoy your usual outdoor activities. [General public]: Enjoy your usual outdoor activities. Moderate 4–6 [at risk]: Adults and children with lung problems, and adults with heart problems, who experience symptoms, should consider reducing strenuous physical activity, particularly outdoors. [General public]: Enjoy your usual outdoor activities. High 7–9 [at risk]: Adults and children with lung problems, and adults with heart problems, should reduce strenuous physical exertion, particularly outdoors, and particularly if they experience symptoms. People with asthma may find they need to use their reliever inhaler more often. Older people should also reduce physical exertion. [General public]: Anyone experiencing discomfort such as sore eyes, cough or sore throat should consider reducing activity, particularly outdoors. Very High 10 [at risk]: Adults and children with lung problems, adults with heart problems, and older people, should avoid strenuous physical activity. People with asthma may find they need to use their reliever inhaler more often. [General public]: Reduce physical exertion, particularly outdoors, especially if you experience symptoms such as cough or sore throat. *Provider:* Local authorities and environmental agencies. *Target:* community/ population. *Channel:* personal via a mobile phone app. *Tailoring:* no | *Self-reported.* Behaviour change: e.g.: On average, how frequently do you change your travel route or route for other outdoor activities because of increased air pollution?' | *'How frequently do you decide not to go outside...?’:* 65.7% (N=46/70) replied ‘never’; 20% (n=14) ‘less than monthly’; 7.1% (n=5) ‘< once a month’; 4.3% (n=3) ‘≥ once a week’; 2.9% (n=2) ‘≥ once a day’. *'Adjust or change your plan for outdoor activities...?’*: 58.6% (n=41) said ‘never’; 24.3% (n=17) ‘less than monthly’; 8.6% (n=6) ‘< once a month’; 5.7% (n=4) ‘≥ once a week’; 2.9% ‘≥ once a day’. *‘Change your travel route...?'* 57.1% (n=40) said ‘never’; 18.6% (n=13) ‘less than monthly’; 10% (n=7) ‘<once a month’; 7.1% (n=5) ‘≥ once a week’; 7.1% ‘≥ once a day’. *'Reschedule travelling or other outdoor activities..?’*: 58.6% (n=41) said ‘never’; 27.1% (n=19) ‘less than monthly’; 5.7% (n=4) ‘< once a month’; 1.4% (n=1) ‘≥ once a week’; 7.1% ‘≥ once a day’. *'Change your means of travel..?*’: 61.4% (n=43) said ‘never’; 21.4% (n=15) ‘less than monthly’; 7.1% ‘<once a month’; 5.7% ‘≥ once a week’; 4.3% (n=3) ‘≥ once a day'. | [Through PCA, 3 components of travel choice were found].  Based on hierarchical regression, **use of the app** did not predict component1 (f1: going out/planning/organising activities outdoors), **explained 4.4% of variance in f2 (f2: attitudes about and frequency of changes in travel time/route)** (B=0.237, p=0.033)**, and 3.2% of variance in f3 (f3: attitude and frequency of changes in choice of means of transport)** (B=0.20, p=0.045)**. Frequency of symptoms predicted f1-f2-f3** (f1, B=0.39, p=0.001; f2, B=0.31, p=0.012, f3, B=0.22, p=0.050)**; Health problems predicted changes in f2 (**B=-0.24, p=0.031)**, beliefs of health impact of air pollution predicted f3** (B=0.28, p=0.007); **use of other sources of information predicted all (**f1, B=0.23, p=0.021; f2, B=0.24, p=0.028, f3, B=0.38, p<0.001), **and higher age predicted only changes in f1** (B=0.24, p=0.019); gender. |
| Licskai et al. (39) | Web browser-based asthma action plan smartphone application (SPA).  Participants transmitted symptoms and peak flow data daily, and received automated control assessment, treatment advice and environmental alerts. Symptoms, β2-agonist use and adherence to asthma control medication were entered daily via the app. Daily AQHI forecasts for the next day were sent at 3pm with corresponding risk reduction message; real-time notification was sent if the AQHI forecast differed significantly from current conditions; e-mail alerts were sent for moderate and high-risk days; and asthma control assessment displayed with relative self-management advice. *Provider:* Health Canada & Environment Canada. *Target:* individuals. *Channel:* the SPA, email reminders of medication adherence. Participants instructed on how to use the app, the AQHI & health advice, and received a written action plan. *Tailoring:* Partial. | *Self-reported.* ‘Yes’ or ‘No’ response to a questions on dose and frequency of use of controller inhaler. Self-reported risk reduction behaviour in response to the AQHI (daily), and self-reported health care use (weekly). | All subjects (100%) reported they used the SPA to determine whether their asthma was controlled, with 86% reporting following action plan recommendations to improve control. 50% changed their behaviour at least once, & 32% changed their behaviour ≥ 6 times in response to a moderate risk AQHI health message such as “consider reducing or rescheduling strenuous activities outdoors if you are experiencing symptoms”. | Not considered. |
| Lyons et al. (36) | The airAware system sent alerts in near real-time alerts.  *Advice:* Air Quality Band Green (≤ 66 for PM10): No message; Yellow (67-106): If you experience symptoms consider reducing strenuous physical activity, particularly outdoors; Amber (107-175): Reduce strenuous physical activity, particularly outdoors. Keep your asthma reliever inhaler with you. Follow your doctor's usual advice about managing your condition; Red (≥176): Avoid strenuous physical activity, particularly outdoors. Keep your inhaler with you. Follow your doctor's usual advice about managing your condition. *Provider*: independent contractor on behalf of the multiagency Local Service Board. *Target:* general public. *Channel:* personal delivery via text, email, pre-recorded voice. *Tailoring:* no. | *Objective health service utilisation due to respiratory symptoms.* The researchers assume that if adherent, participants should use hospital less frequently. | Intervention associated with a statistically significant increase in emergency admissions for respiratory conditions (IRR: 3.97; 95% CI [1.59- 9.93]) and A&E attendance (IRR=1.89; 95% CI [1.34-2.68]). No statistically significant increase in GP contacts between groups, or prescribed medications, or out-patients attendances. | Not considered. |
| Mak, et al. (43) | The Hong Kong Air pollution index (API) is derived from RSP, SO_2_, CO, O_3_ and NO_2_ concentrations.    *Advice:* when “very high” levels, people with heart or respiratory illness should avoid prolonged stay in areas with heavy traffic. Low (0-25) Not expected health impact. Medium (26-50) Not expected for the general public. High (51-100) Few or none in the general public may notice immediate health effects. Long term effects may be observed if exposed to such levels persistently for a long time. Very High (101-200) People with exiting heart or respiratory illnesses may notice mild aggravation of their health conditions. Healthy individuals may also notice some discomfort. Severe (201-500) People with exiting heart or respiratory illnesses may experience significant aggravation of their symptoms and there will be also widespread symptoms in the healthy population. *Provider:* Local authority. *Target:* general public. *Channel:* various (not stated). *Tailoring:* no | *Self-reported.* Response to high API: possible answers: ‘Limit the time for outdoor activities’; ‘Keep my kids at home from school’; ‘Take the leave from work; wear a mask’; ‘Tell other people about the health risk’; ‘Get advice from doctor’. | Only 117 respondents (17.7% amongst the n=660 people who heard of API) took actions when the API was at high level. Of the actions taken: ‘Limited the time for outdoor activities’ was mostly chosen (n=115), ‘Wear a mask’ (n=42), ‘Tell other people about the risk’ (n=31), ‘Get advice from doctor’ (n=11), ‘Take leave from work’ (n=10), ‘Keep my kids at home from school’ (n=7). | T-Test: being a person who spends most of their working time at busy streets vs. the general public. **Age: being** **≥60 year old;** with a significant difference between age groups (p<0.0001); LSD test confirmed the only significant difference existed between interviewees aged <19 (8.49%, n=9/106 samples aged < 9 had ever taken actions) compared to people aged ≥ 60 (31.11% took action), [p not reported]. |
| Sugerman et al. (31) | Emergency risk communications about air pollution during San Diego wildfire in 2007.  *Advice:* Protection from smoke inhalation. Nontechnical messages included for example “stay indoors,” “keep windows and doors closed,” and “only exercise indoors”; and messages using technical terminology were: “use N95 respirators during clean-up,” “use HEPA air filters,” and “run air conditioners on recirculate.” *Provider:* the San Diego County Health and Human Services Agency, with the American Heart and Lung Associations. *Target:* Community of San Diego. *Channel:* via television, radio, newspaper, & the Internet. Delivery source as self-reported: TV n=1146 (77.1%); Radio n=96 (6.5%); Internet n=20 (1.3%); Other n=224 (15.1%). *Tailoring:* No | *Self-reported.* Adherence to risk messages. A general Q: ‘Thinking now about all of the messages that you saw or heard during the week of the fires and that applied to you, would you say you followed all, most, some, or none of those messages? Plus specific Qs, e.g.: Did you keep all windows and doors completely closed? Did you wear a mask? | *Global message compliance* (the degree of compliance among all messages heard, saw, understood, and applicable to them): Adherence to all messages, n=444 (26.8%); Most messages, n=887 (53.6%); Some n=221 (13.3%); Very few n=73 (4.4%); None n=31 (1.9%). People stayed inside their homes most of the day (≥ 5 days) (n=1047, 58.7%), kept all windows and doors closed (≥ 5 days) (n=1339, 75.8%), did not participate in outdoor sports (0 days) (n=1580, 88.4%), and wet ash with water before and during home clean-up (records considered n=925, n=693, 75.6%). Fewer respondents used home air conditioning (n=267, 15.5%), HEPA air filtration (≥ 5 days) (n=181, 10.3%), or wore N95 respirators during ash clean-up (n considered 925, n=75, 8.1%). Of those with asthma 31.5% increased use of metered inhaler, whilst 41.3% and 23.7% of those with COPD increased respectively use of inhaler and oxygen flow rates. | *Adherence to most/all messages:* **female** [n=697/830 (84.0%) vs. male: n=634/826 (76.8%), p <.001]; **spoke English as primary language** [n=1113/ 1360 (81.8%) vs. Spanish/other n=211/286 (73.8%), p= .003]**; higher education** [n=1061/1298 (81.7%) vs. ≤High school n=261/348 (75.0%), p= .006], **incomes of ≥$50,000** [n=704/842 (83.6%) vs. <$50,000 n=419/549 (76.3%), p <.001]**; higher exposure to smoky air** [3–7 days (81.5%) vs. 0–2 days (75.9%), p=.025]**, a history of asthma or COPD** [Yes n=231/272 (84.9%) vs. No n=1100/1384 (79.5%), p= .045]**, reporting depression** [Yes n=370/439 (84.3%) vs. No n=961/1217 (79.0%) p=.017]; age; ethnicity; employment; increased inhaler use; heart failure; saw doctor during fires. By univariate linear regression, **higher average of messages complied with: [for non-technical & technical messages]: age group 18–64 year-old** vs. 65+ (respectively M=2.0 vs. M=1.7; M=0.9 vs. M=0.7; all p<.001); **employed full time** vs. part-time (M=2.0 vs. M=1.9, p=0.048; M=0.9 vs. M=0.8, p=0.001; p=0.001)**, with more than 2 days of exposure** vs. 3-7 days (M=1.6 vs. M=2.2; M=0.2 vs. M=1.8; all p<0.001)**; those with asthma or COPD** vs. without (M=2.1 vs. M= 1.9, p=0.002; M=1.0 vs. M= 0.8, p<0.001; p<0.001)**, persons reporting increased inhaler use** vs. no increase (M=2.3 vs. M= 1.9, p=0.013; M=1.2 vs. M= 0.8, p<0.001; p<0.001)**, reporting depression during the fires** vs. no depression (M=2.2 vs. M= 1.9, p<0.001; M=1.0 vs. M= 0.8, p<0.001). More-technical messages were recalled and followed less than non-technical ones (p values not reported). |
| **INTENDED ADHERENCE** | | | | |
| Johnson (27) | The Pollutant Standard Index (PSI) was tested: i.e. 5 versions combining 3 elements: old and new descriptors, recommendation to limit outdoor exertion for sensitive groups, and definition of sensitive groups. The PSI presented 4 hypothetical 'index values' for the day, which were 2 above (i.e. 110 & 160) and 2 below (i.e. 60 & 80) the standard.  *Advice:* (details in Johnson (2003) (27). *Provider:* US Environmental Protection Agency (EPA). *Target:* general public. *Channel:* handouts with alternative written format of the PSI for the scopes of the study. *Tailoring:* No | *Self-reported.* Level of agreement with the following: 'Given this information, I expect in future to be active outdoors on days when this level of air pollution occurs. | 47% (overall n=1038 used for this analysis) agreed or strongly agreed with the statement about their intention to be active outdoors [including both above and below standard indices]. | After reading the PSI, **white women and non-white women** compared to non-white men [respectively 41% and 43% reported the intention to be active outdoors, compared to 54% of non-white men, p<0.05 (analysis for non-white women no longer significant after Bonferroni correction at p<.0085)]. **Being an English speaker** (pre-PSI 72% of n=130 non-English-speakers vs. 46% of n=890 English-speakers at home reported to be active for ≥4 h, p<00001; whilst Post-PSI 56% of n=125 Non-English speakers vs. 45% of n=855 English speakers intended to be active outdoors, p<0.05)^b^. When analysing only the groups reading the above standard PSI, being English speakers was no longer significant. |
| Johnson (28) | *Advice:* V.1: the Old version: Old Descriptors: Good (up to 50), Moderate (50-100), Unhealthful (100-200), Very Unhealthful (200-300). V.2: Old Descriptors, Health Cautions: e.g.: 'Increasing likelihood of respiratory symptoms and breathing discomfort in sensitive groups' & Cautionary Statement: 'Health notice: sensitive children and adults and people with respiratory disease, such as asthma, should limit prolonged, moderate exertion outdoors'. V.3: New Descriptors: Good (up to 50), Moderate (51-100), Unhealthy for Sensitive Groups (101-150), Generally Unhealthy (151-200), Very Unhealthy (201-300). V.4: New Descriptors & Sensitive Groups definition: e.g., 'active children, outdoor workers, and people with respiratory disease, such as asthma'. V.5: new descriptors, Health Cautions & Sensitive Group. V. 6 (control group): no PSI. *Provider:* US EPA. *Target:* general public. *Channel:* handouts with alternative PSI formats, for the scopes of the study. *Tailoring:* No | *Self-reported.* Level of agreement with the following: 'Given this information, I expect in future to be active outdoors on days when this level of air pollution occurs. | *When above-standard indices presented:* 43% of Old format readers (N=145), & 50% of New format readers agreed with the statement about intention to be active outdoors (n=155); Whilst 52% of readers of the Cautious format (n=448) vs 43% of the non-Cautious version (n=590) agreed; finally, 48% (n=442) of those reading the definition of Sensitive groups vs. 45% (n=596) of the no-definition group were expecting to be active during high pollution. | *When above-standard indices presented:* **being an Old format reader** (43% vs. 50% of New format readers agreed with the statement about intention to be active outdoors during pollution, Zadj=2.07, p<0.05); **reading the no-Cautious format** (52% of readers of the Cautious format vs 43% of the no-Cautious version agreed, Zadj=2.42, p<0.05); reading a definition of Sensitive groups (48% reading the format with the definition agreed vs. 45% of the no-definition group (Zadj=0.28, p>0.05). |
| Smallbone (37) | UK Air Quality Index alert (2010).  *Advice:* Air Quality index (former version): Low: Effects are unlikely to be noticed even by individuals who know they are sensitive to air pollutants; Moderate: Mild effects, unlikely to require action, may be noticed amongst sensitive individuals. High: Significant effects may be noticed by sensitive individuals and action to avoid or reduce these effects may be needed (e.g. reducing exposure by spending less time in polluted areas outdoors). Asthmatics will find that their 'reliever' inhaler is likely to reverse the effects on the lung. Very High: The effects on sensitive individuals described for 'High' levels of pollution may worsen. *Provider:* Local authorities and environmental agencies. *Target:* general public. *Channel:* general news media, text messages or email for subscribers, however for the survey a specific AQI presented as part of an online survey. *Tailoring:* No | *Self-reported.* Respondents were asked if they would consider taking action in response to an air quality index alert and if so, what kind of action would they take. [exact index level presented to participants, not reported] | Overall intended adherence: 36.9%. *Most popular behaviour change by all participants (n=206) (participants could select ≥ 1):* avoid locations that people thought to be polluted (39%, n=80); 24% (n=50) stay indoors more or keep preventative medication near (27%, n=56); 22% (n=46) stated they could not change; 15% (n=30) would not change as the information was not important to them. | *Distribution in subgroups:* 57% of those with a respiratory condition (n=88) intended to take action, 36% (n=32) would not/could not; 7% answered 'don't know'. In the healthy group: 66% would not change, 21% answered 'yes' and 13% did not know'. *What change?* Those with a respiratory condition: 64% (n=53/83) would ensure easy access to reliever medications or take a preventative dose (30%, n=25); 45% (n=37) would avoid ‘polluted areas’, 41% (n=34) would stay ‘indoors more or all day’, (24%) would avoid strenuous exercise, & 6% would alter exercise location. In the healthy group: 36% (n=42/118) would avoid ‘polluted areas’, 14% (n=16) would alter ‘exercise location’, 3% would avoid strenuous exercise, 13% would stay indoors more or all day, 2% would use preventative inhaler. |

Notes:

a. In bold are reported statistically significant results.

b. However, the pre-PSI measure reported current behaviour, whilst the post-PSI measure reported intended behaviour referred to a specified high level of pollution.

Definitions:

CO=carbon monoxide; NO_2_=nitrogen dioxide; O_3_=ozone; PM_2.5_ = fine particulate matter; RSP= respirable suspended particulates; SO_2_ = sulphur dioxide; TRI =Toxic Release Inventory; TRS=total reduced sulphur.
